# *L*-citrulline and *L*-arginine improve reproductive performance in primiparous and multiparous cows via distinct shared metabolic pathways and pyrimidine metabolism

**DOI:** 10.3389/fvets.2025.1659668

**Published:** 2025-10-22

**Authors:** Chen Fan, Changzheng Chen, Renping Liu, Muredili Maimaiti, Changgeng Li, Weijie Zao, Waike Lv, Guozhu Xu, Guodong Zhao

**Affiliations:** ^1^College of Animal Science, Xinjiang Agricultural University, Urumqi, China; ^2^Karamay Lvcheng Agricultural Development Co., Ltd., Karamay, China; ^3^Xinjiang Agricultural Net Animal Husbandry Co., Ltd., Bole, China; ^4^Boertala Mongolian Autonomous Prefecture Animal Disease Control and Diagnosis Center, Bole, China

**Keywords:** *L*-Arg, *L*-Cit, cows, reproductive performance, early embryo apoptosis

## Abstract

**Background:**

To investigate the impact of *L*-arginine (*L*-Arg) and *L*-citrulline (*L*-Cit) on cow reproductive performance.

**Methods:**

90 healthy, age-matched Simmental cows were randomly assigned to three groups: control, *L*-Arg, and *L*-Cit, with 30 cows per group. Estrus synchronization was performed, and various parameters were assessed, including estrus rate, conception rate, and early embryo apoptosis, following intraperitoneal administration of *L*-Arg and *L*-Cit. Additionally, reproductive hormones, serum antioxidant levels, and plasma metabolites were measured.

**Results:**

Results indicated that both the estrus and conception rates in the *L*-Arg and *L*-Cit groups were significantly higher than those in the control group (*p* < 0.05). Furthermore, serum LH levels and VEGF concentrations on day 1 and day 8 were elevated in the *L*-Arg and *L*-Cit groups compared to controls (*p* < 0.05). Estrus rate and serum NO concentration in the *L*-Cit group were notably higher than those in the control group (*p* < 0.05). At day 8 and day 21, estrus rate, conception rate, P levels, and serum VEGF concentrations were significantly greater in both the *L*-Arg and *L*-Cit groups compared to controls (*p* < 0.05). Additionally, serum NO concentration was significantly higher in the *L*-Cit group than in the control group (*p* < 0.05). The activities of T-AOC and GSH-Px in serum were significantly increased in both primiparous and multiparous cows in the *L*-Arg and *L*-Cit groups relative to the control group (*p* < 0.05). Metabolomic analysis revealed that in primiparous cows, the protein digestion and absorption, ABC transporters, and AMPK signaling pathways were significantly enriched. In multiparous cows, pyrimidine metabolism was notably enriched.

**Conclusion:**

Under the experimental conditions, L-Arg modulated the production of Dimethyl sulfone, 1,5-pentanediamine, Bassanolide, and Phenylalanine in primiparous cows through the protein digestion and absorption and ABC transporters pathways, while *L*-Cit influenced the same metabolites *via* the ABC transporters pathway. Both *L*-Arg and *L*-Cit promoted increased NO, GSH-Px, T-AOC, and VEGF levels, enhancing early embryo implantation. Additionally, *L*-Arg and *L*-Cit synergistically influenced pyrimidine metabolism, regulating the production of N-myristoylphosphorylcholine, N-oleoyl-d-erythro-sphingosylphosphorylcholine, 1-stearoyl-2-docosahexaenoyl-sn-glycero-3-phosphocholine, and 1-docosahexaenoyl- 2-stearoyl-sn-glycero-3-phosphocholine. This modulation resulted in increased NO, GSH-Px, T-AOC, and VEGF in multiparous cows, ultimately supporting early embryo implantation.

## 1 Introduction

The application of Ovsynch-TAI in cattle breeding has become well-established, yet challenges persist due to suboptimal feeding management and imbalanced nutrition, leading to negative nutritional status in cows. This imbalance results in a lack of estrus, absence of ovulation, reduced estrus rates, and low conception rates during estrus synchronization, ultimately prolonging breeding cycles and increasing breeding costs. Recent research highlights the critical role of nutrition regulation in animal reproduction, particularly in managing estrus delays, cycle disruptions, reduced pregnancy rates, and early embryonic development. Carvalho found that nutritional regulation can optimize the reproductive performance of cows (1). Additionally, certain amino acids, which are vital for nutrition, also offer significant potential in improving reproductive function and efficiency in cows (2). *L*-arginine (*L*-Arg), a conditionally essential amino acid, plays multiple physiological roles in mammals, while *L*-citrulline (*L*-Cit) serves as its precursor. As a precursor to Nitric oxide (NO), *L*-Arg positively influences follicular development by promoting angiogenesis and anti-apoptosis within follicles (3). The Arg/NO pathway stimulates GnRH and LH secretion at the hypothalamic–pituitary level, thereby enhancing follicular development and ovulation (4). Moreover, *L*-Arg has been shown to improve endothelial function, increase fetal-placental blood flow, and stimulate placental angiogenesis. Studies have indicated that *L*-Arg enhances reproductive performance in female sheep and improves embryo survival rates (5). Furthermore, research on non-pregnant ewes demonstrated that short-term *L*-Arg supplementation positively impacted the development of large and small follicles at various stages (6). An injection of 155 μmol/kg *L*-Cit during fetal development was also shown to increase follicle numbers in ewes (7). Building on estrus synchronization, this study investigates the effects of intraperitoneal injection of *L*-Arg and *L*-Cit on estrus rate, pregnancy rate, serum reproductive hormones, early embryo apoptosis, antioxidant capacity, and metabolic levels in cows, offering a theoretical foundation for the use of *L*-Arg and *L*-Cit in cow estrus synchronization.

## 2 Materials and methods

### 2.1 Test time and place

The experiment was carried out in Xinjiang Agricultural Network Animal Husbandry Co., Ltd.in Bortala Hot Spring County, Xinjiang from June 2022 to July 2023 (longitude: 81.606239, latitude: 44.993801).

### 2.2 Test instruments and materials

#### 2.2.1 Experimental animals

A total of 180 Simmental cows were selected, including 90 primiparous and 90 multiparous cows. After examination, all cows were found to be healthy, with well-developed reproductive systems and no reproductive-related diseases. All cows were screened by vaginal mucus microscopy (no pathogenic bacteria) and B-ultrasound (no ovarian cysts or uterine inflammation) to confirm no reproductive disorders. Body weight and body condition scores (BCS) were recorded for all cows. The body weight and BCS data are expressed as mean ± standard deviation, with the basic information provided in Table 1.

**Table 1:** Basic information of cows.

Item	Primiparous cattle			Multiparous cattle		
	Control group	*L*-Arg group	*L*-Cit proup	Control group	*L*-Arg group	*L*-Cit proup
Body weight	360.80 ± 35.73	357.50 ± 36.03	363.77 ± 36.19	387.70 ± 31.31	392.47 ± 29.26	376.87 ± 33.39
BCS	5.36 ± 0.79	5.37 ± 0.75	5.37 ± 0.83	5.33 ± 0.92	5.30 ± 0.75	5.23 ± 0.89

#### 2.2.2 Test drugs

*L*-Arg (purity ≥ 99%) was purchased from Aladdin Reagent (Shanghai) Co., Ltd., and *L*-Cit (purity ≥ 99%) was purchased from Hebei Hongtao Bioengineering Co., Ltd. Gonadorelin for injection (GnRH, 200 μg/mL) was sourced from Ningbo Sansheng Co., Ltd., while cloprostenol injection (PG, 0.2 mg/mL) was purchased from Ningbo Second Hormone Factory. Cow early pregnancy colloidal gold test strips were provided by Beijing Wanhua Bioengineering Co., Ltd. The dG · Blue Eyes bovine pregnancy rapid diagnosis visual kit was sourced from Beijing Xiangzhong Biotechnology Co., Ltd. The enzyme-linked immunosorbent assay kit was purchased from Shanghai Enzyme-linked Biotechnology Co., Ltd. Other laboratory materials included acetonitrile (Fisher), methanol (Fisher), ultrapure water (Fisher), and liquid injection bottles (Agilent). The chromatographic column parameters were 1.7 μm, 2.1 × 100 mm (Waters).

#### 2.2.3 Test equipment

The equipment included a microplate reader (iMark, Bio-Rad), veterinary B-mode ultrasonic instrument (Kaixin RKU 10, Xuzhou Kaixin Technology Co., Ltd.), AB Triple TOF 6600 mass spectrometer (AB SCIEX), Q Exactive HF-X mass spectrometer (Thermo), Q Exactive HF mass spectrometer (Thermo), Agilent 1,290 Infinity LC ultra-high pressure liquid chromatograph (Agilent), Vanquish UHPLC ultra-high pressure liquid chromatograph (Thermo), low-temperature high-speed centrifuge (Eppendorf 5430R), insemination gun, vacuum blood collection tube, electronic balance, gloves, cellulose acetate filter, disposable syringes, and disposable long-arm gloves.

### 2.3 Feeding management

Throughout the experiment, all cows were provided a standardized diet, fed twice daily (at 8:00 and 19:00), and given access to free drinking water. The nutritional composition and levels of the diet are shown in Table 2.

**Table 2:** Diet composition and nutrient level (dry matter basis) %.

Component	Content	Component	Content
TMR raw materials		Nutrition level of^2^	
Fodder	20	Moisture	7.89
Green storage	36	Crude protein	12.60
Yellow storage	26	Crude fat	2.87
Maize	15	Coarse ash	11.77
Cottonseed meal	2	Acid detergent fiber	17.62
Premix^1^	1	Neutral detergent fiber	29.55
Total	100	Total phosphorus	0.47
		Sulfur	0.14
		Dry matter	92.11
		Ca	0.99
		Na	0.21
		Cl	0.56
		K	1.16
		Mg	0.29

1 Premix: VA 14000 IU, VD3 40,000 IU, VE 600 IU, copper 200 mg, iron 1,500 mg, manganese 1,000 mg, zinc 1,200 mg, iodine 100 mg, selenium 30 mg, cobalt 6 mg, nicotinic acid 200 mg.

2 The nutritional levels were measured.

### 2.4 Experimental design

A total of 180 healthy Simmental cows, comprising 90 primiparous and 90 multiparous cows, were selected for the experiment. Both primiparous and multiparous cows were divided into two batches for simultaneous treatment, with 45 cows in each batch. These cows were randomly assigned to three groups: control, *L*-Arg, and *L*-Cit, with 15 cows in each group. All cows were raised under identical feeding conditions with free access to water. The first injection of GnRH was administered on day 0 of the experiment.

In the control group, cows received an intramuscular injection of GnRH and an intraperitoneal injection of normal saline (80 mL/head) on day 0, followed by an intramuscular injection of PG (3 mg) and an intraperitoneal injection of normal saline (80 mL/head) on day 7. On day 9, an intramuscular injection of GnRH and an intraperitoneal injection of normal saline (80 mL/head) were administered. Artificial insemination was performed 14 h after the GnRH injection.

The *L*-Arg group received an intramuscular injection of GnRH and an intraperitoneal injection of *L*-Arg solution (155 μmol/kg) on day 0, followed by an intramuscular injection of PG and an intraperitoneal injection of *L*-Arg solution (155 μmol/kg) on day 7. On day 9, an intramuscular injection of GnRH and an intraperitoneal injection of *L*-Arg solution (155 μmol/kg) were administered, followed by artificial insemination 14 h later.

The *L*-Cit group was given an intramuscular injection of GnRH and an intraperitoneal injection of *L*-Cit solution (155 μmol/kg) on day 0, an intramuscular injection of PG and an intraperitoneal injection of *L*-Cit solution (155 μmol/kg) on day 7, and an intramuscular injection of GnRH and an intraperitoneal injection of *L*-Cit solution (155 μmol/kg) on day 9, followed by artificial insemination at a 14-h interval.

Day 0: intramuscular injection of GnRH (200 μg/head); day 7: intramuscular injection of PG (0.2 mg/head); day 9: intramuscular injection of GnRH (200 μg/head).

### 2.5 Solution preparation and intraperitoneal injection method

To prepare the *L*-Arg and *L*-Cit solutions, the required amounts of *L*-Arg and *L*-Cit were weighed and dissolved in sterile normal saline (0.9% sodium chloride) to achieve a concentration of 155 μmol/kg body weight (8). The pH of the solution was adjusted to 7.0 using sodium hydroxide or hydrochloric acid, and the solution was filtered through a 0.22 μm cellulose acetate filter. The prepared solution was stored in a sterile container for use. The total injection volume for each cow was 80 mL of either *L*-Arg or *L*-Cit solution.

For intraperitoneal injection, the cow was positioned in a Baoding head restraint, and the injection site was located on the right flank. After shearing and disinfecting the area, a No. 16 needle was inserted perpendicular to the skin, piercing the abdominal muscle and peritoneum. When the needle passed through the peritoneum, resistance decreased, and a sense of “give” was felt. At this point, the infusion tube was connected, and the solution was injected into the abdominal cavity. The syringe was slowly pressed to control the flow rate during injection (9).

### 2.6 Sample collection and processing

Blood samples were collected from the tail root vein of the cows on the 1st, 8th, 10th, 21st, and 28th days of the experiment (6 cows were randomly selected from each group on days 1, 8, and 10, while all test cows were sampled on days 21 and 28 after artificial insemination). In order to prevent the degradation of metabolites, blood samples were centrifuged within 30 min after collection; the serum was centrifuged and packed into a 3 mL cryopreservation tube within 1 h and stored in a liquid nitrogen environment for subsequent analysis.

### 2.7 Preparation of serum samples

On the 28th day post-artificial insemination, the serum was slowly thawed at 4 °C. A portion of the serum was added to a pre-cooled methanol/acetonitrile/water solution (2:2:1, v/v), vortexed, mixed, and subjected to low-temperature ultrasound for 30 min. The mixture was then allowed to stand at −20 °C for 10 min and centrifuged at 14,000 g at 4 °C for 20 min. The supernatant was vacuum-dried for further analysis. For mass spectrometry, the dried sample was reconstituted by adding 100 μL of a 1:1 (v/v) acetonitrile/water solution, vortexed, and centrifuged at 14,000 g at 4 °C for 15 min. The supernatant was then collected for analysis.

The chromatographic column used was the Waters ACQUITY UPLC BEH Amide 1.7 μm, 2.1 × 100 mm. The mobile phase consisted of Phase A (ultrapure water with 25 mM ammonium acetate and 25 mM ammonia) and Phase B (acetonitrile). The flow rate was set to 0.5 mL/min, the column temperature was maintained at 40 °C, and the injection volume was 2 μL. A liquid-phase elution gradient was employed for separation.

For mass spectrometry, the electrospray ion source temperature was set to 650 °C, and the mass spectrum voltage was 5,500 V for positive ions and −4,500 V for negative ions. The decluster voltage was set at 60 V, with ion source gas 1 and gas 2 at 60 psi, and the gas curtain pressure at 30 psi. The ionization parameters were set to high impact-induced conditions.

### 2.8 Determination of antioxidant and hormone levels in serum

The determination of antioxidant and hormone levels in serum was carried out following the instructions of the kit, and a microplate reader was used for detection.

### 2.9 Data statistics and analysis

Excel 2018 software was used for initial data sorting. The estrus rate, conception rate, and early embryo apoptosis data were analyzed using the chi-square test in SPSS 26.0. One-way analysis of variance (ANOVA) was employed to assess hormone levels, vascular endothelial growth factor (VEGF), and antioxidant levels.

The original metabolomics data were converted into mzXML format using ProteoWizard, followed by peak alignment, retention time correction, and peak area extraction using XCMS software. The data extracted from XCMS were subjected to metabolite structure identification and preprocessing. The quality of the experimental data was then evaluated, and subsequent data analysis was performed, including univariate statistical analysis, multidimensional statistical analysis, differential metabolite screening, correlation analysis of differential metabolites, and KEGG pathway analysis.

## 3 Results and analysis

### 3.1 Effects of intraperitoneal injection of *L*-Arg and *L*-Cit on estrus rate, return to estrus rate and conception rate of cows

As shown in Table 3, the estrus rate(ER) in the control group was 36.7% (11/30), while the estrus rates in the *L*-Arg and *L*-Cit groups were 70.0% (21/30) and 63.3% (19/30), respectively. Compared to the control group, the estrus rate in the *L*-Arg group increased by 33.3% (*p* < 0.05), and in the *L*-Cit group, it increased by 26.6% (*p* < 0.05). The return rate in the control group was 27.2% (3/11), in the *L*-Arg group it was 42.9% (9/21), and in the *L*-Cit group, it was 42.1% (8/19). No significant differences were observed in the return rate among the three groups (*p* > 0.05). The pregnancy rate(PR) in the control group was 26.7% (8/30), while the pregnancy rate in the *L*-Arg and *L*-Cit groups were 63.3% (19/30) and 50.0% (15/30), respectively. Compared to the control group, the pregnancy rate in the *L*-Arg group increased significantly by 36.6% (*p* < 0.05). Although the pregnancy rate in the *L*-Cit group increased by 23.3%, the difference was not statistically significant (*p* > 0.05).

**Table 3:** Effects of intraperitoneal injection of L-Arg and L-Cit on estrus rate, estrus rate and conception rate of cows.

Item	Primiparous cattle			Multiparous cattle		
	Control group	*L*-Arg group	*L*-Cit group	Control group	*L*-Arg group	*L*-Cit group
Estrus rate	36.7%^b^	70.0%^a^	63.3%^a^	60.0%^b^	80.0%^ab^	93.3%^a^
Return rate	27.2%	42.9%	42.1%	27.8%	29.2%	21.4%
Pregnancy rate	26.7%^b^	63.3%^a^	50.0%^ab^	56.7%^b^	80.0%^ab^	93.3%^a^

In the same column, values with different lowercase superscript letters mean significant difference (*p* < 0.05), different uppercase superscript letters mean extremely significant difference (*p*<0.01), while with the same or no superscript letters mean no significant difference (*p* > 0.05).

The estrus rate in the control group was 60.0% (18/30), while in the *L*-Arg and *L*-Cit groups, it was 80.0% (24/30) and 93.3% (28/30), respectively. Compared to the control group, the estrus rate in the *L*-Cit group was significantly higher, with an increase of 33.3% (*p* < 0.05). The pregnancy rate in the control group was 56.7% (17/30), while in the *L*-Arg and *L*-Cit groups, it was 80.0% (24/30) and 93.3% (28/30), respectively. Compared to the control group, the pregnancy rate in the *L*-Cit group was significantly higher by 36.6% (*p* < 0.05). Although the pregnancy rate in the *L*-Arg group increased by 23.3%, the difference was not statistically significant (*p* > 0.05).

### 3.2 Effects of intraperitoneal injection of L-Arg and L-Cit on the hormones in the serum of cows

**Figure 1:**
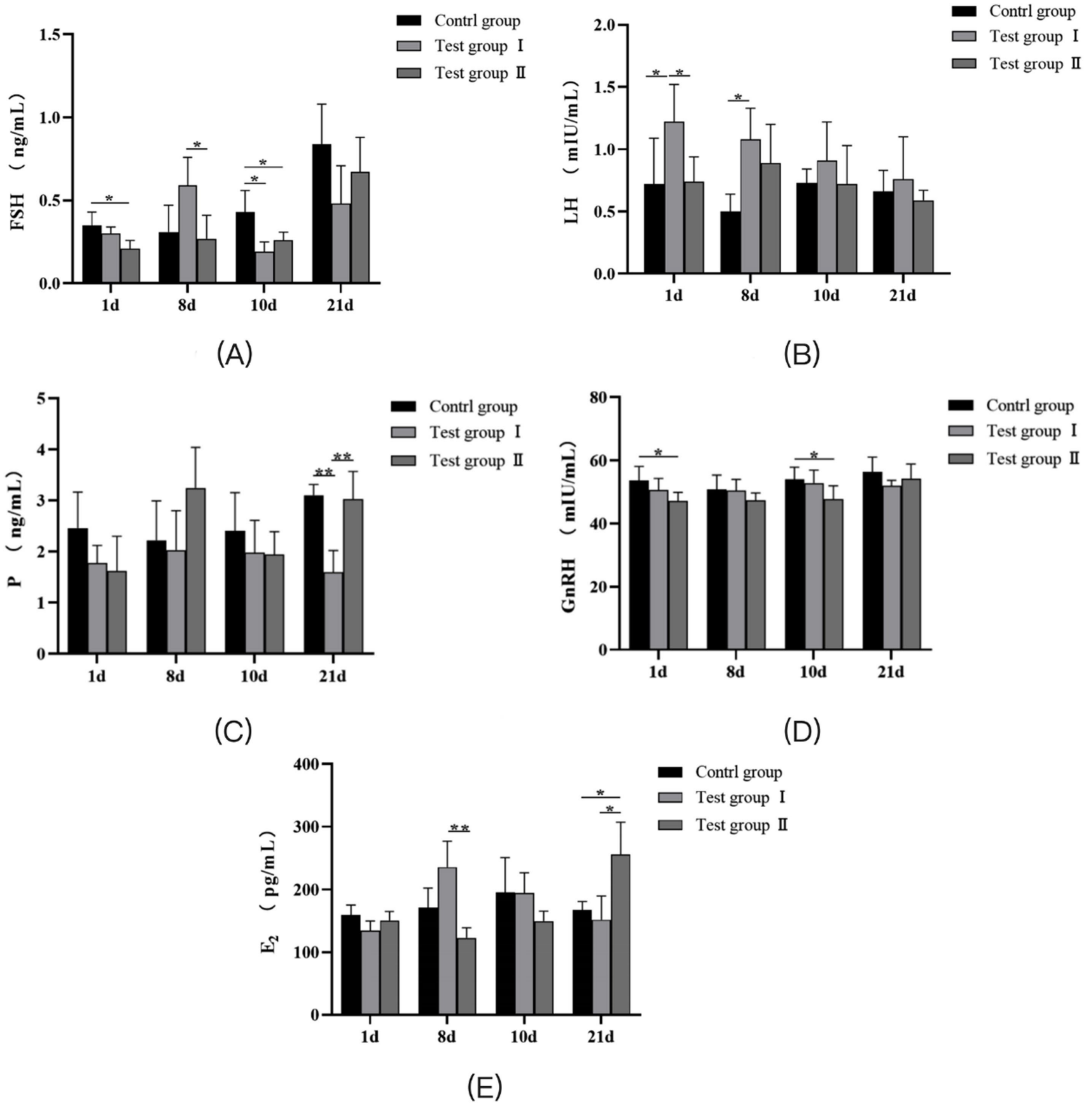
Effects of intraperitoneal injection of L-Arg and L-Cit on serum hormones in primiparous cows. **(A)** FSH levels in primiparous cows at different times. **(B)** LH level of primiparous cows at different time. **(C)** P level of primiparous cows at different time. **(D)** GnRH level of primiparous cows at different time. **(E)** E2 level of primiparous cows at different time. Test group I was intraperitoneally injected with L-Arg group, and Test group II was intraperitoneally injected with L-Cit group. In the figure, ‘*’ and ‘**’ indicated that the difference between the groups was significant (*p* < 0.05) or extremely significant (*p* < 0.01), and no label indicated that the difference was not statistically significant (*p* > 0.05).

As shown in Figure 1A, the serum FSH content in the *L*-Cit group of primiparous cows on the 1st and 10th days was significantly lower than that in the control group (*p* < 0.05). On the 8th day, the Follicle-Stimulating Hormone(FSH) content in the serum of the *L*-Arg group was significantly higher than that of the *L*-Cit group (*p* < 0.05), but significantly lower than that of the control group (*p* < 0.05). On the 10th day, no significant differences in FSH content were observed among the three groups (*p* > 0.05). As presented in Figure 1B, the serum Luteinizing Hormone(LH) content in the *L*-Arg group of primiparous cows was significantly higher than that in the control group on days 1 and 8 (*p* < 0.05). On days 10 and 21, there was no significant difference in LH content between the three groups (*p* > 0.05). As shown in Figure 1C, on the 21st day, the serum Progesterone(P) content in the *L*-Arg group was significantly lower than that in the control and *L*-Cit groups (*p* < 0.01). No significant differences in P content were observed among the three groups on days 1, 8, and 10 (*p* > 0.05). Figure 1D indicates that the serum Gonadotropin-releasing Hormone(GnRH) content in the *L*-Cit group of primiparous cows was significantly lower than that in the control group on days 1 and 10 (*p* < 0.05). There was no significant difference in GnRH content between the three groups on days 8 and 21 (*p* > 0.05). According to Figure 1E, the serum E2 content in the *L*-Arg group of primiparous cows was significantly higher than that in the *L*-Cit group on the 8th day (*p* < 0.01). On the 21st day, the serum Estradiol (E_2_) content in the *L*-Cit group was significantly higher than that in the control and *L*-Arg groups (*p* < 0.05). No significant differences in E2 content were observed between the three groups on days 1 and 10 (*p* > 0.05).

**Figure 2:**
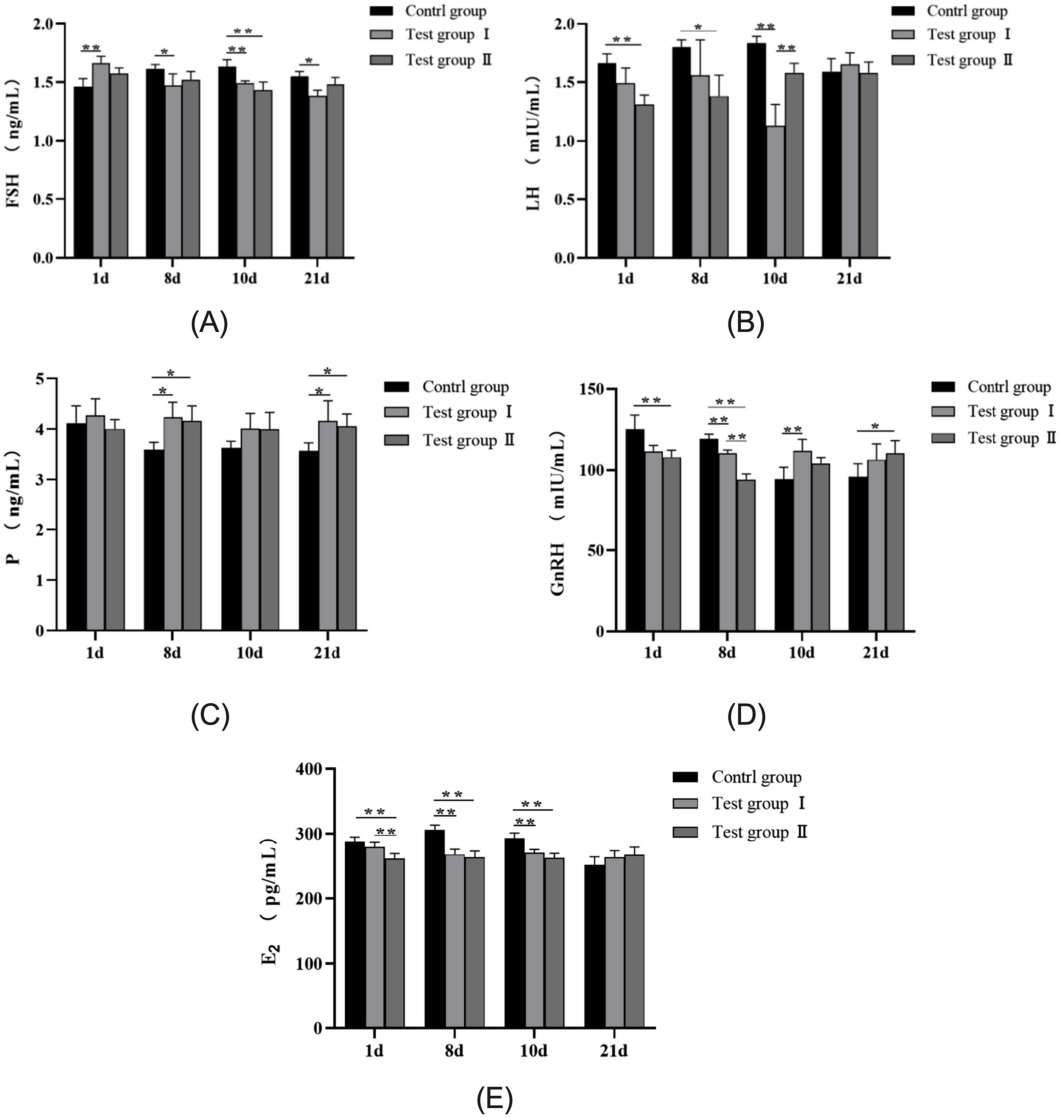
Effects of intraperitoneal injection of *L*-Arg and *L*-Cit on serum hormones in multiparous cows. **(A)** FSH levels in multiparous cows at different times. **(B)** LH levels in multiparous cows at different times. **(C)** P level of multiparous cows at different time. **(D)** GnRH level of multiparous cows at different time. **(E)** E2 level of multiparous cows at different time.

As shown in Figure 2A, serum FSH levels in the *L*-Arg group of multiparous cows were significantly higher than in the control group on day 1 (*p* < 0.01). Conversely, serum FSH levels in the control group were significantly higher than in the *L*-Arg group on days 8 and 21 (*p* < 0.05). On day 10, serum FSH levels in the control group exceeded those in both the *L*-Arg and *L*-Cit groups (*p* < 0.01). Figure 2B illustrates that serum LH levels in the control group were significantly higher than in the *L*-Cit group on days 1 and 8 (*p* < 0.05). On day 10, serum LH levels in the *L*-Arg group were significantly lower than in both the control and *L*-Cit groups (*p* < 0.01). No significant difference in LH levels was observed among the three groups on day 21 (*p* > 0.05). Figure 2C reveals that serum P levels in the control group were significantly lower than in both the *L*-Arg and *L*-Cit groups on days 8 and 21 (*p* < 0.05). As shown in Figure 2D, serum GnRH levels in the control group were significantly higher than in the *L*-Cit group on days 1 and 8 (*p* < 0.01). On day 10, serum GnRH levels in the control group were significantly lower than in the *L*-Arg group (*p* < 0.01). On day 21, serum GnRH levels in the control group were significantly lower than in the *L*-Cit group (*p* < 0.05). Figure 2E shows that serum E2 levels in the *L*-Cit group were significantly lower than in both the control and *L*-Arg groups on day 1 (*p* < 0.01). On days 8 and 10, serum E2 levels in the control group were significantly higher than in both the *L*-Arg and *L*-Cit groups (*p* < 0.01). No significant difference in E2 levels was observed among the three groups on day 21 (*p* > 0.05).

### 3.3 Effects of intraperitoneal injection of *L*-Arg and *L*-Cit on early embryonic apoptosis in cows

Table 4 shows that, compared to the control group, the 21-day conception rate in the *L*-Arg and *L*-Cit groups of primiparous cows increased by 16.6 and 13.3%, respectively, while the 28-day conception rate rose by 23.4 and 10.0%. The 42-day conception rate increased by 36.6 and 23.3%, respectively, but no significant differences were observed between the groups (*p* > 0.05). Regarding early embryo apoptosis, from days 21 to 28, the apoptosis rate decreased by 15.0 and 1.7% in the *L*-Arg and *L*-Cit groups, respectively. From days 28 to 42, apoptosis rates dropped by 23.4 and 22.2%, respectively. Over the entire period (21 to 42 days), the apoptosis rate decreased by 36.0% in the *L*-Arg group and 22.5% in the *L*-Cit group, but no significant differences were noted between the groups (*p* > 0.05).

**Table 4:** Effects of intraperitoneal injection of *L*-Arg and *L*-Cit on early embryo apoptosis in cows.

Item	Time	Primiparous cattle			Multiparous cattle		
		Control group	*L*-Arg group	*L*-Cit group	Control group	*L*-Arg group	*L*-Cit group
Pregnancy rate	21 day	66.7%	83.3%	80.0%	76.7%^b^	93.3%^ab^	96.7%^a^
	28 day	43.3%	66.7%	53.3%	63.3%^b^	83.3%^ab^	93.3%^a^
	42 day	26.7%	63.3%	50.0%	56.7%^b^	80.0%^ab^	93.3%^a^
Apoptosis Rates	21–28 day	35.0%	20.0%	33.3%	17.3%	10.7%	3.4%
	28–42 day	28.4%	5.0%	6.25%	10.5%	4.0%	0%
Total apoptosis rate	21–42 day	60.0%	24.0%	37.5%	26.1%^a^	14.2%^ab^	3.4%^b^

Apoptosis rate = (Conception rate at earlier time point–Conception rate at later time point)/Conception rate at earlier time point × 100%. That the decrease in conception rate is mainly due to early embryo apoptosis. 21–28 days apoptosis rate = (21 days conception rate-28 days conception rate)/21 days conception rate × 100%; 28–42 days apoptosis rate = (28 days conception rate-42 days conception rate)/28 days conception rate × 100%; 21–42 days apoptosis rate = (21 days conception rate-42 days conception rate)/21 days conception rate × 100%. In the same column, values with different lowercase superscript letters mean significant difference (*p* < 0.05), different uppercase superscript letters mean extremely significant difference (*p*<0.01), while with the same or no superscript letters mean no significant difference (*p* > 0.05).

In multiparous cows, the conception rate in the *L*-Arg group increased by 16.6, 20.0, and 23.3% on days 21, 28, and 42, respectively, but no significant difference was observed (*p* > 0.05). In the *L*-Cit group, the conception rate increased by 20.0% (*p* < 0.05), 30.0% (*p* < 0.05), and 36.6% (*p* < 0.05) on days 21, 28, and 42, respectively. Early embryo apoptosis in the *L*-Arg and *L*-Cit groups decreased by 6.6 and 13.9%, respectively, from days 21 to 28, and by 6.5 and 10.5% from days 28 to 42, but no significant differences were observed (*p* > 0.05). However, from days 21 to 42, the *L*-Cit group showed a significant decrease in early embryo apoptosis by 22.7% (*p* < 0.05), while the *L*-Arg group exhibited a non-significant reduction of 11.9% (*p* > 0.05).

### 3.4 Effects of intraperitoneal injection of *L*-Arg and *L*-Cit on NO and VEGF in cows

**Figure 3:**
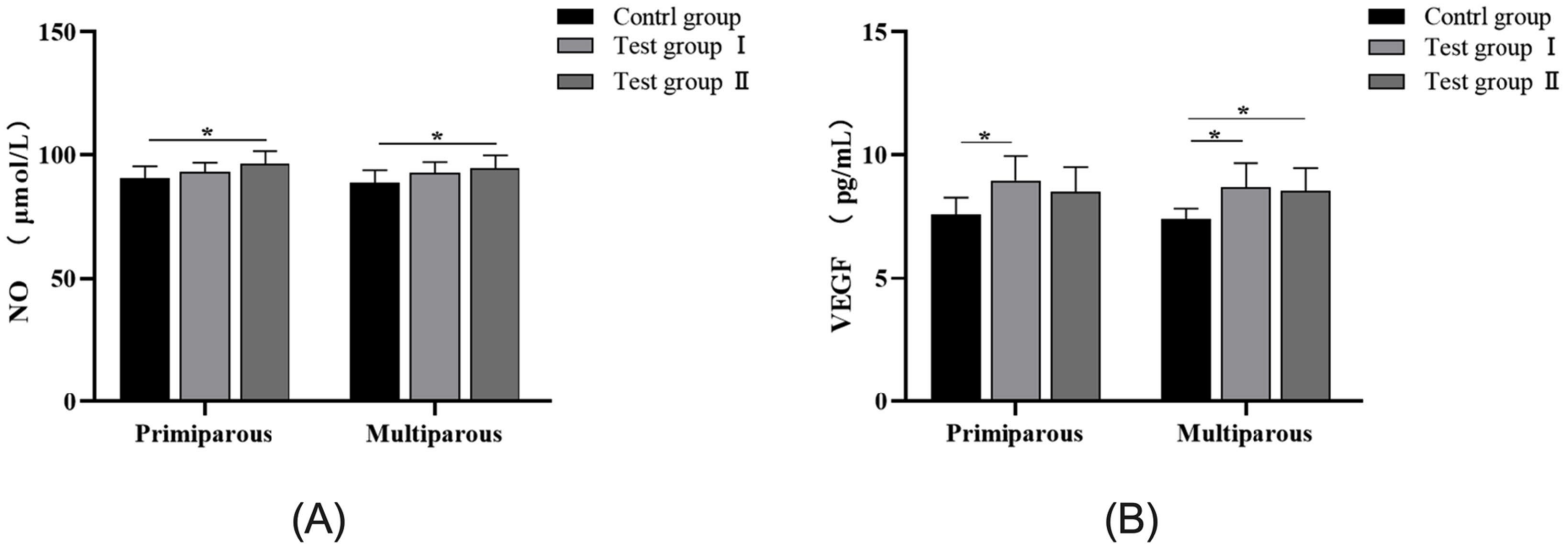
Effects of intraperitoneal injection of *L*-Arg and *L*-Cit on NO and VEGF in cows. Error bars represent standard error of the mean (SEM), *n* = 6 per group. **(A)** NO levels in multiparous cows at different times. **(B)** VEGF levels in multiparous cows at different times.

As can be seen from Figure 3, the concentrations of nitric oxide (NO) and vascular endothelial growth factor (VEGF) in the serum of cows showed an upward trend after intraperitoneal injection of *L*-Arg and *L*-Cit. For primiparous cows, the concentration of NO in the *L*-Cit group was significantly higher than that in the control group (*p* < 0.05), and there was no significant difference in the concentration of NO between the *L*-Arg group and the control group (*p* > 0.05). The concentration of VEGF in the *L*-Arg group was significantly increased by 15.4% compared with that in the control group (*p* < 0.05), and the concentration of VEGF in the *L*-Cit group was increased by 10.8% compared with that in the control group (*p* > 0.05). For multiparous cows, the concentration of NO in the *L*-Cit group was significantly higher than that in the control group (*p* < 0.05), and there was no significant difference in the concentration of NO between the *L*-Arg group and the control group (*p* > 0.05). The concentrations of VEGF in the *L*-Arg group and the *L*-Cit group were increased by 14.9% (*p* < 0.05) and 13.4% (*p* < 0.05) respectively compared with that in the control group.

### 3.5 Effects of intraperitoneal injection of *L*-Arg and *L*-Cit on the antioxidant level of cows

**Figure 4:**
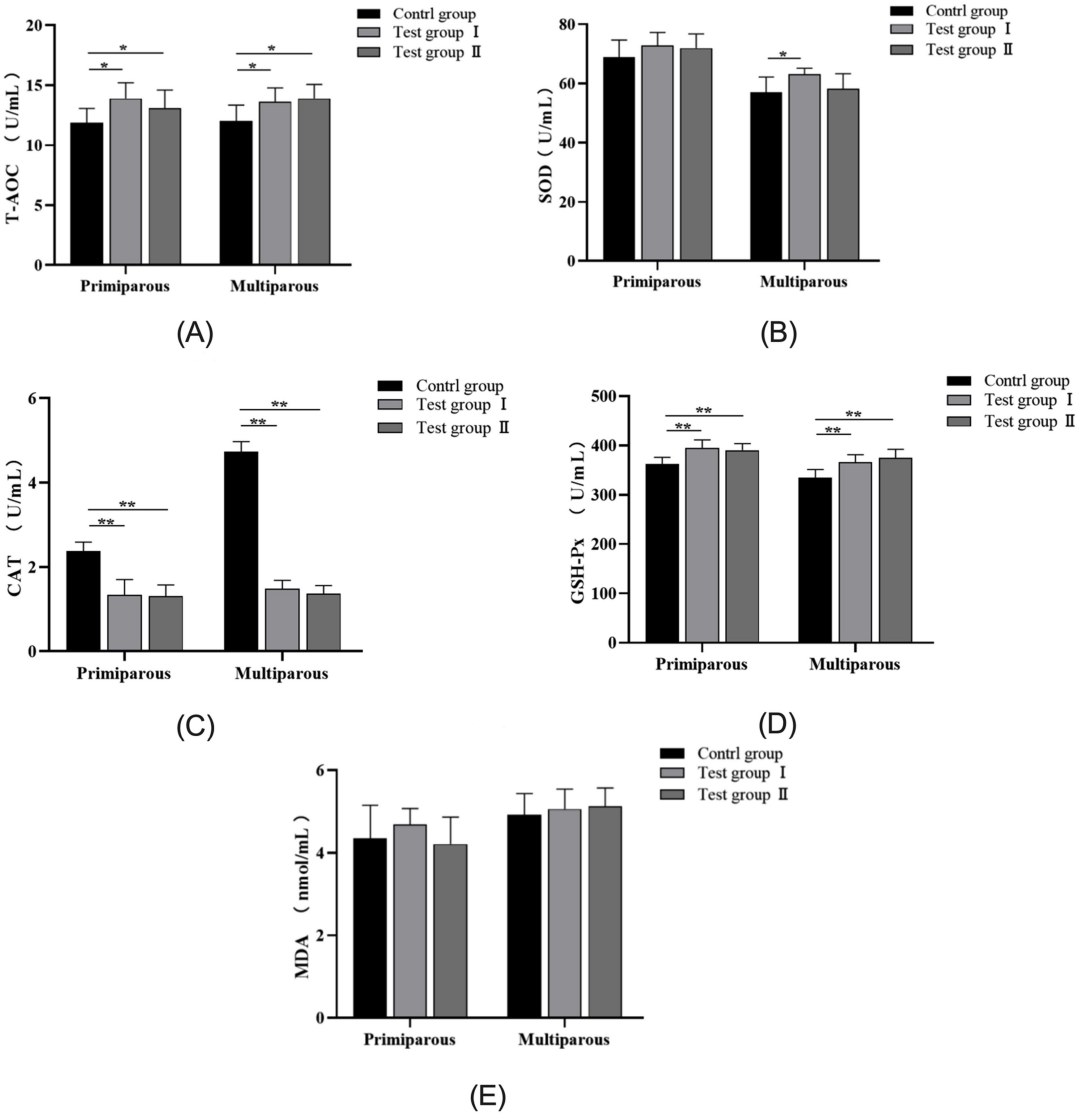
Effects of intraperitoneal injection of *L*-Arg and *L*-Cit on the antioxidant level of cows. Error bars represent standard error of the mean (SEM), *n* = 6 per group. **(A)** T-AOC levels in multiparous cows at different times. **(B)** LH levels in multiparous cows at different times. **(C)** P level of multiparous cows at different time. **(D)** GnRH level of multiparous cows at different time. **(E)** E2 level of multiparous cows at different time.

As can be seen from Figure 4, for primiparous cows, the Total antioxidant capacity(T-AOC) concentration in serum from the *L*-Arg and *L*-Cit groups increased by 14.6% (*p* < 0.05) and 9.5% (*p* < 0.05), respectively, compared to the control group. The concentration of Catalase (CAT) decreased by 43.4% (*p* < 0.01) and 44.7% (*p* < 0.01), while Glutathione peroxidase(GSH-Px) levels increased by 8.3% (*p* < 0.01) and 7.1% (*p* < 0.01), respectively, in the *L*-Arg and *L*-Cit groups. Superoxide Dismutase (SOD) concentrations increased by 5.5% (*p* > 0.05) and 4.3% (*p* > 0.05), with no significant difference observed. No significant differences in Malondialdehyde (MDA) concentration were noted among the three groups (*p* > 0.05).

In multiparous cows, serum T-AOC concentrations in the *L*-Arg and *L*-Cit groups increased by 11.7% (*p* < 0.05) and 13.4% (*p* < 0.05), respectively. The CAT concentration decreased by 68.7% (*p* < 0.01) and 71% (*p* < 0.01), while GSH-Px levels increased by 8.7% (*p* < 0.01) and 10.7% (*p* < 0.01). The concentration of SOD increased by 9.6% (*p* < 0.05) and 2.1% (*p* > 0.05). MDA concentrations increased by 2.8% (*p* > 0.05) and 3.9% (*p* > 0.05), respectively, in the *L*-Arg and *L*-Cit groups compared to the control group.

### 3.6 Effects of intraperitoneal injection of *L*-Arg and *L*-Cit on serum metabolites in cows

#### 3.6.1 Hierarchical clustering analysis

**Figure 5:**
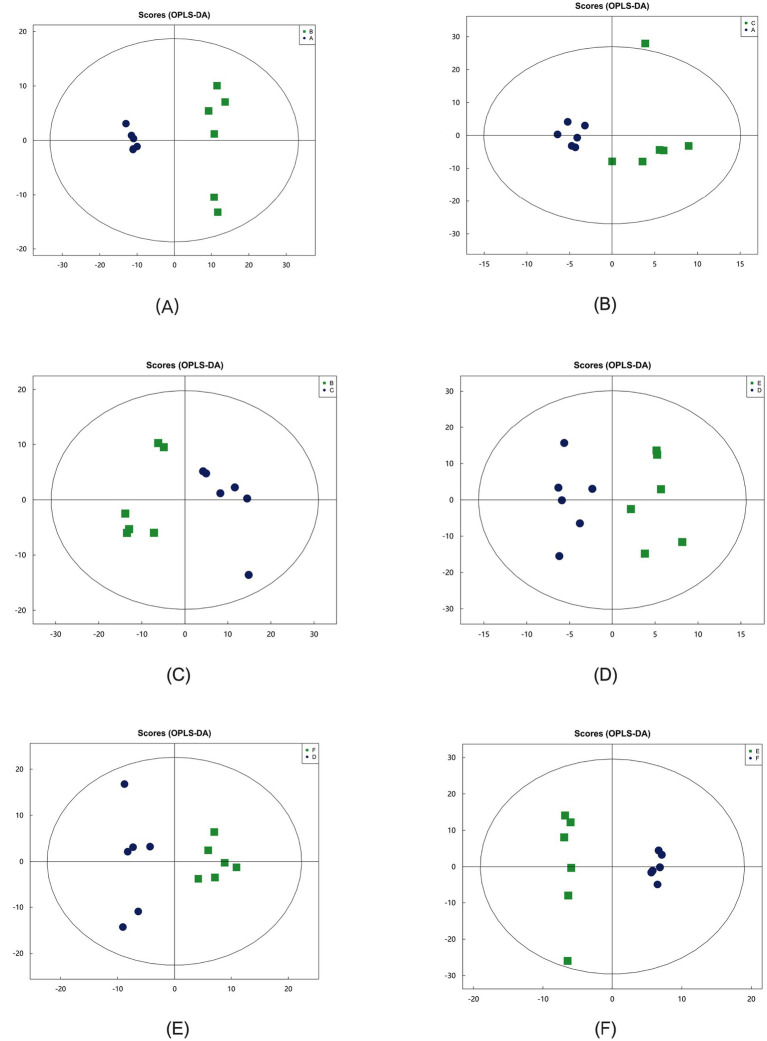
Orthogonal partial least squares discriminant analysis (OPLS-DA) between groups. **(A)**: Primiparous cattle control group; **(B)** *L*-Arg group of primiparous cattle; **(C)** *L*-Cit group of primiparous cattle; **(D)** multiparous cattle control group; **(E)** multiparous cattle *L*-Arg group; **(F)** Multiparous cattle *L*-Cit group.

**Figure 6:**
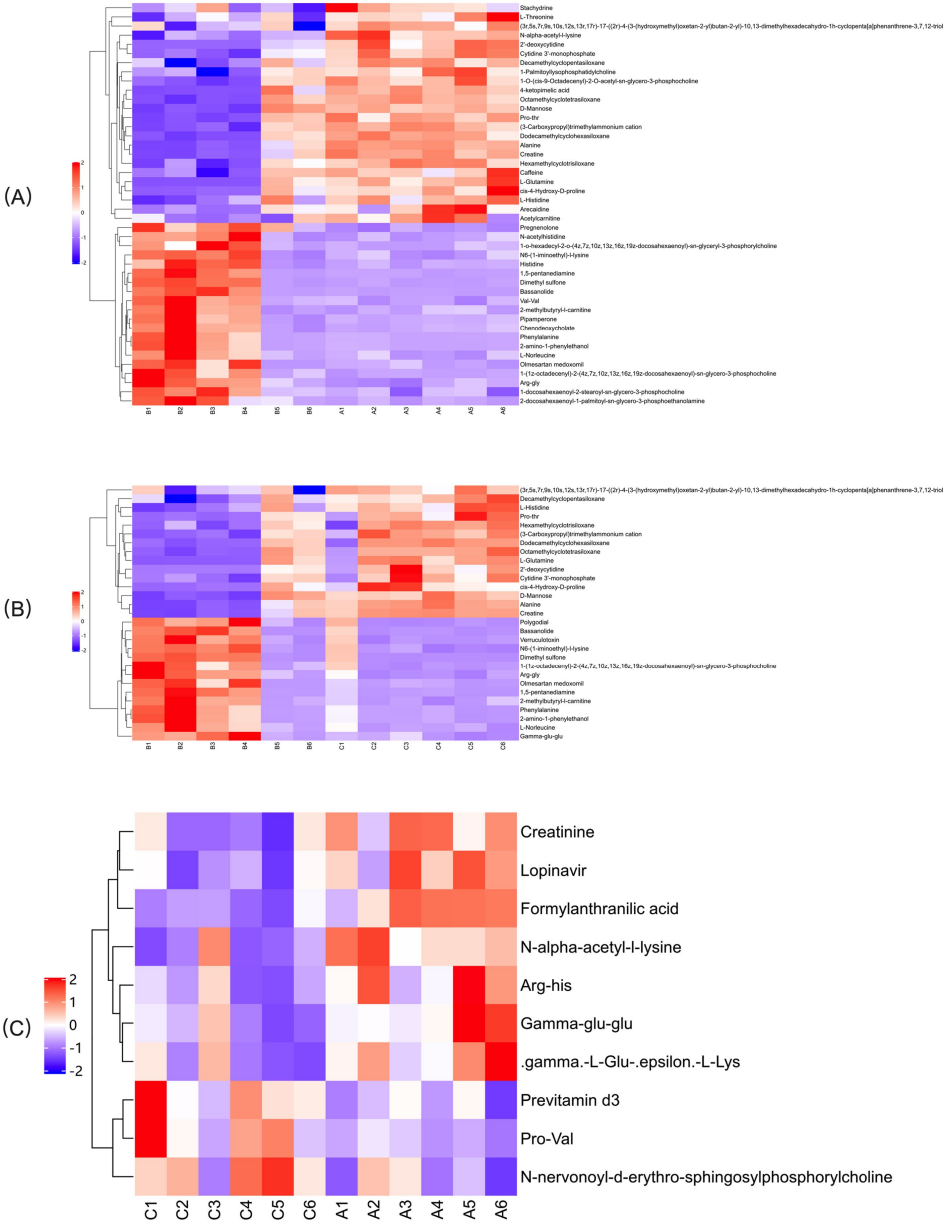
Hierarchical cluster analysis heat map of each comparison combination of primiparous cattle. **(A)** Primiparous cattle control group; **(B)** *L*-Arg group of primiparous cattle; **(C)** Primiparous cattle *L*-Cit group.

**Figure 7:**
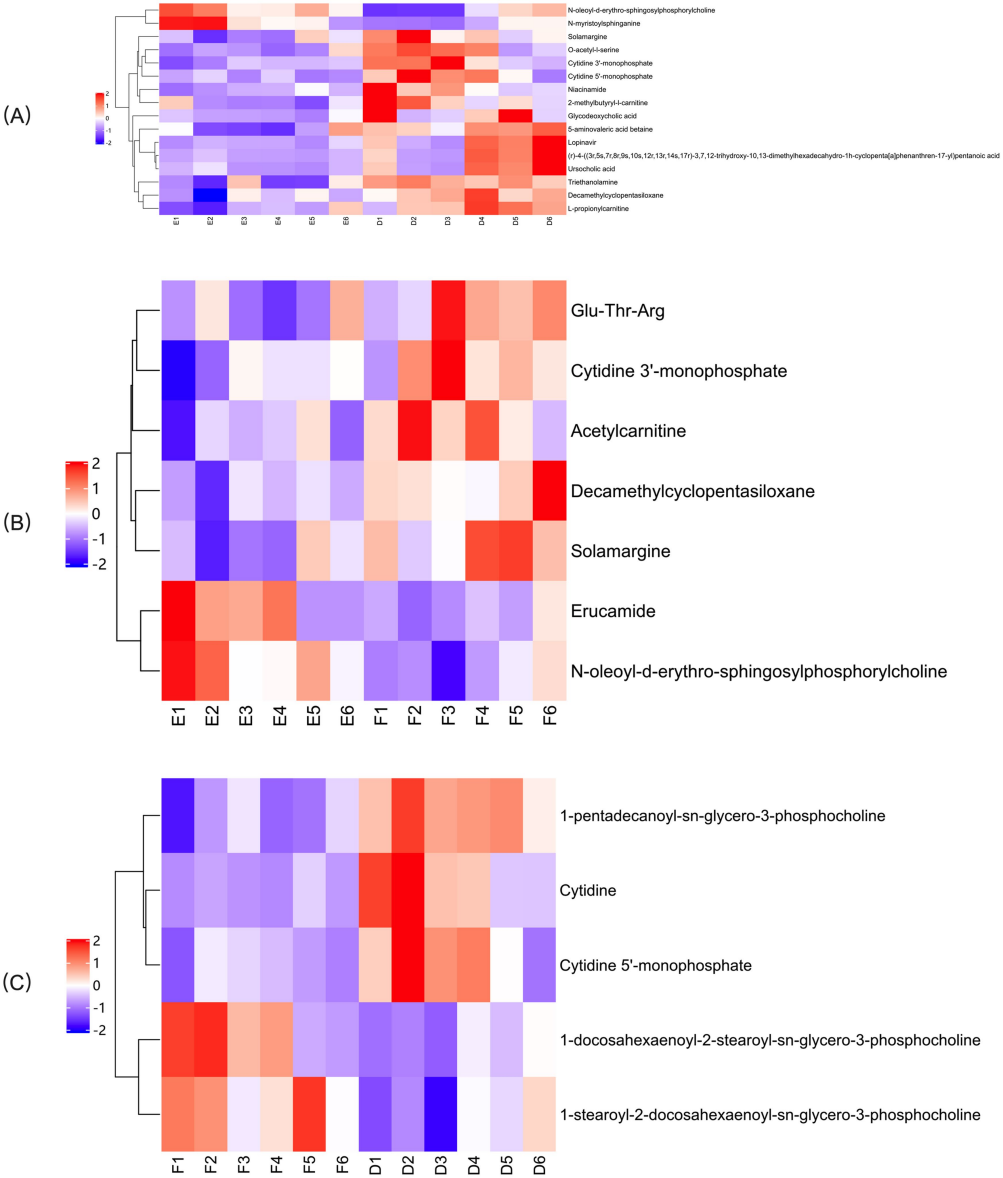
Hierarchical cluster analysis heat map of each comparison combination of multiparous cattle. **(A)** Multiparous cattle control group; **(B)** multiparous cattle *L*-Arg group; **(C)** Multiparous cattle *L*-Cit group.

**Figure 8:**
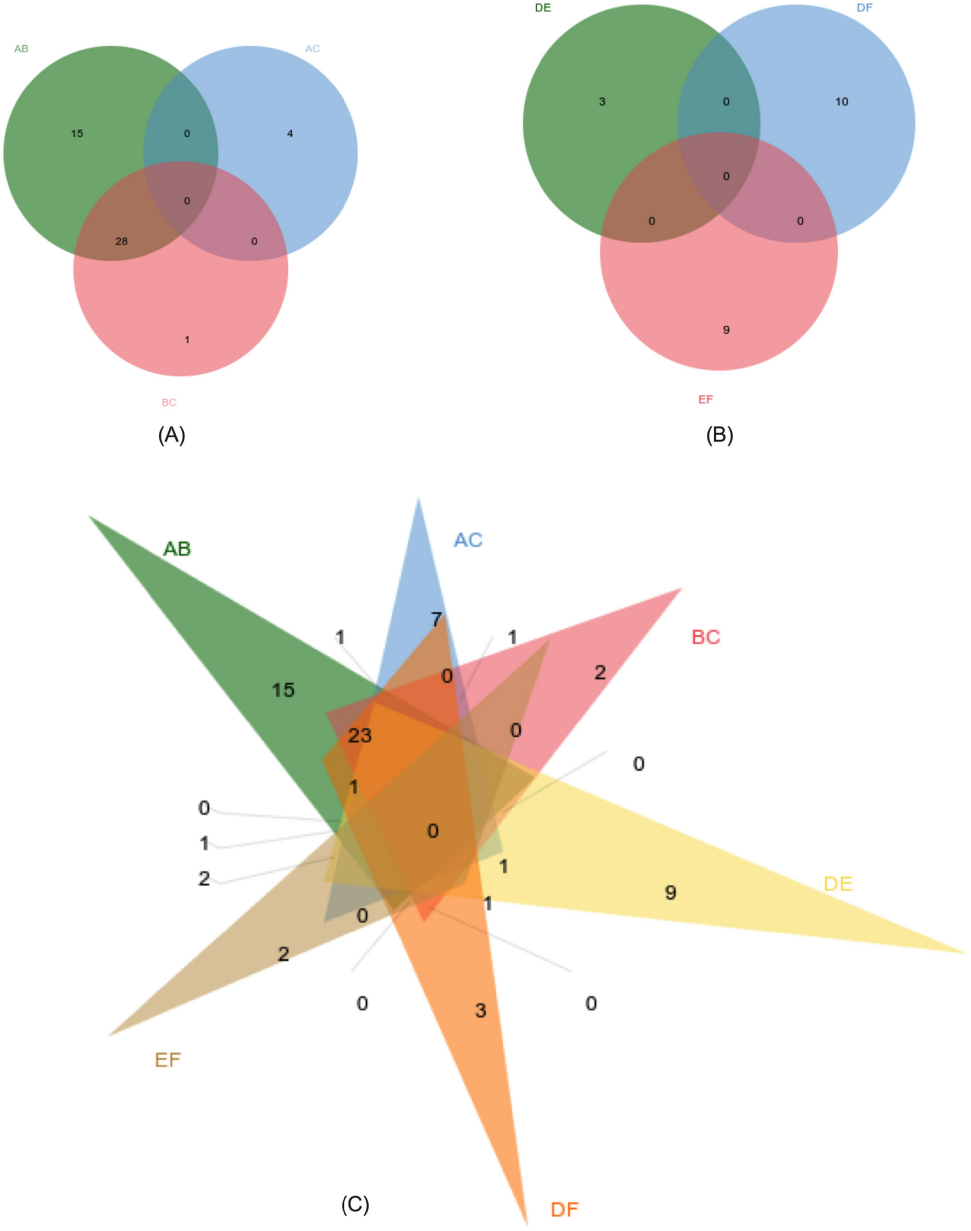
Metabolite co-expression venn diagram. **(A)** Primiparous cattle control group; **(B)** *L*-Arg group of primiparous cattle; **(C)** *L*-Cit group of primiparous cattle; **(D)** multiparous cattle control group; **(E)** multiparous cattle *L*-Arg group; **(F)** Multiparous cattle *L*-Cit group.

In the OPLS-DA model, significant differences were observed in the first principal component scores of serum samples from each group, with all samples falling within the 95% confidence interval (Figure 5). Cluster analysis revealed distinct groupings for the primiparous cattle control group, intraperitoneal injection of *L*-Arg and *L*-Cit groups, as well as the multiparous cattle control group and the corresponding *L*-Arg and *L*-Cit groups, providing a solid basis for subsequent analyses (Figures 6, 7). A total of 48 metabolites were annotated in the control, *L*-Arg, and *L*-Cit groups, with 44 differential metabolites identified between the control and *L*-Cit groups, and 33 differential metabolites identified between the control and *L*-Arg groups. Additionally, 15 specific metabolites were annotated in the *L*-Arg group compared to the control group, 4 specific metabolites in the *L*-Cit group compared to the control group, and 1 differential metabolite in the *L*-Cit group compared to the *L*-Arg group. Furthermore, 28 differential metabolites were common between the control and *L*-Cit groups (Figure 8A). A total of 22 metabolites were annotated across all groups, with 12 differential metabolites identified between the control and *L*-Cit groups, and 19 between the control and *L*-Arg groups. Specific metabolites identified were 3 in the *L*-Arg group compared to the control group, 10 in the *L*-Cit group compared to the control group, and 9 differential metabolites between the *L*-Cit and *L*-Arg groups (Figure 8B). There were 15 differential metabolites between the primiparous cattle control group and the *L*-Arg group, 7 differential metabolites between the primiparous cattle control group and the *L*-Cit group, 2 differential metabolites between the primiparous cattle L-Arg group and the *L*-Cit group, 9 differential metabolites between the multiparous cattle control group and the *L*-Arg group, 3 differential metabolites between the multiparous cattle control group and the *L*-Cit group, and 2 differential metabolites between the multiparous cattle *L*-Arg group and the *L*-Cit group.

#### 3.6.2 Screening of serum differential metabolites

**Figure 9:**
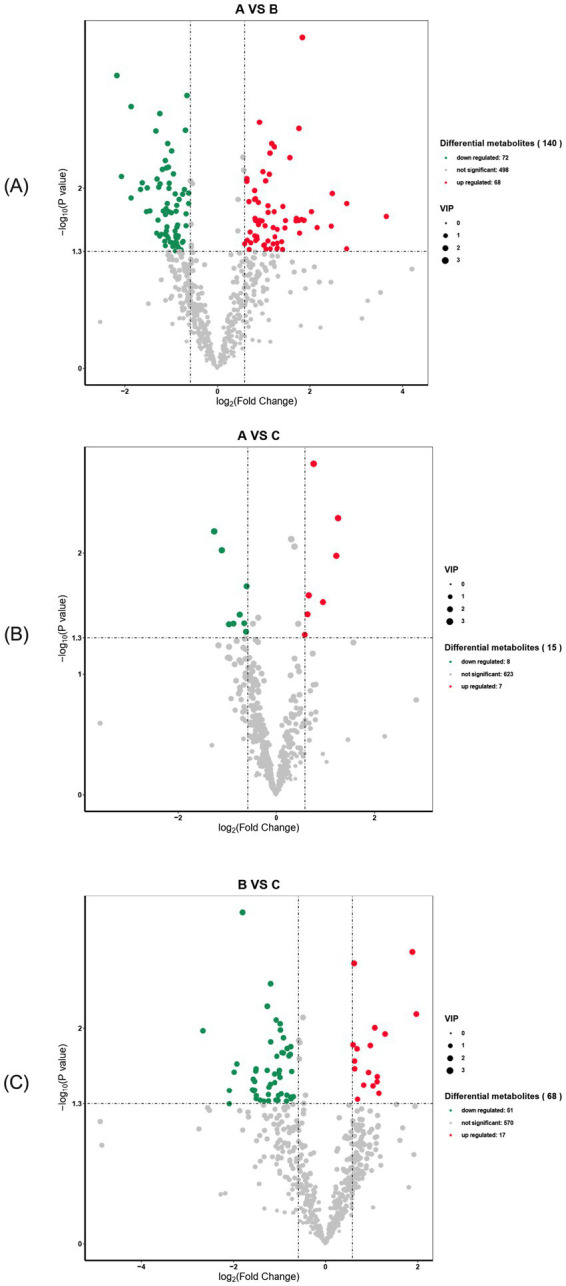
Volcano diagram of differential metabolites in primiparous cattle. **(A)** Primiparous cattle control group VS *L*-Arg group of primiparous cattle; **(B)** Primiparous cattle control group VS *L*-Cit group of primiparous cattle; **(C)** *L*-Arg group of primiparous cattle VS Primiparous cattle *L*-Cit group.

In serum samples from the primiparous cattle control group and the *L*-Arg group, a total of 638 differential metabolites were detected, with 140 showing significant changes. Of these, 72 were down-regulated (*p* < 0.05), 68 were up-regulated (*p* < 0.05), and 498 showed no significant change (Figure 9A). In serum samples from the control and *L*-Cit groups, 638 differential metabolites were detected, with 15 showing significant changes—8 down-regulated (*p* < 0.05), 7 up-regulated (*p* < 0.05), and 623 unaffected (Figure 9B). In serum samples from the *L*-Arg and *L*-Cit groups, 638 differential metabolites were detected, with 68 showing significant changes—51 down-regulated (*p* < 0.05), 17 up-regulated (*p* < 0.05), and 570 unchanged (Figure 9C).

**Figure 10:**
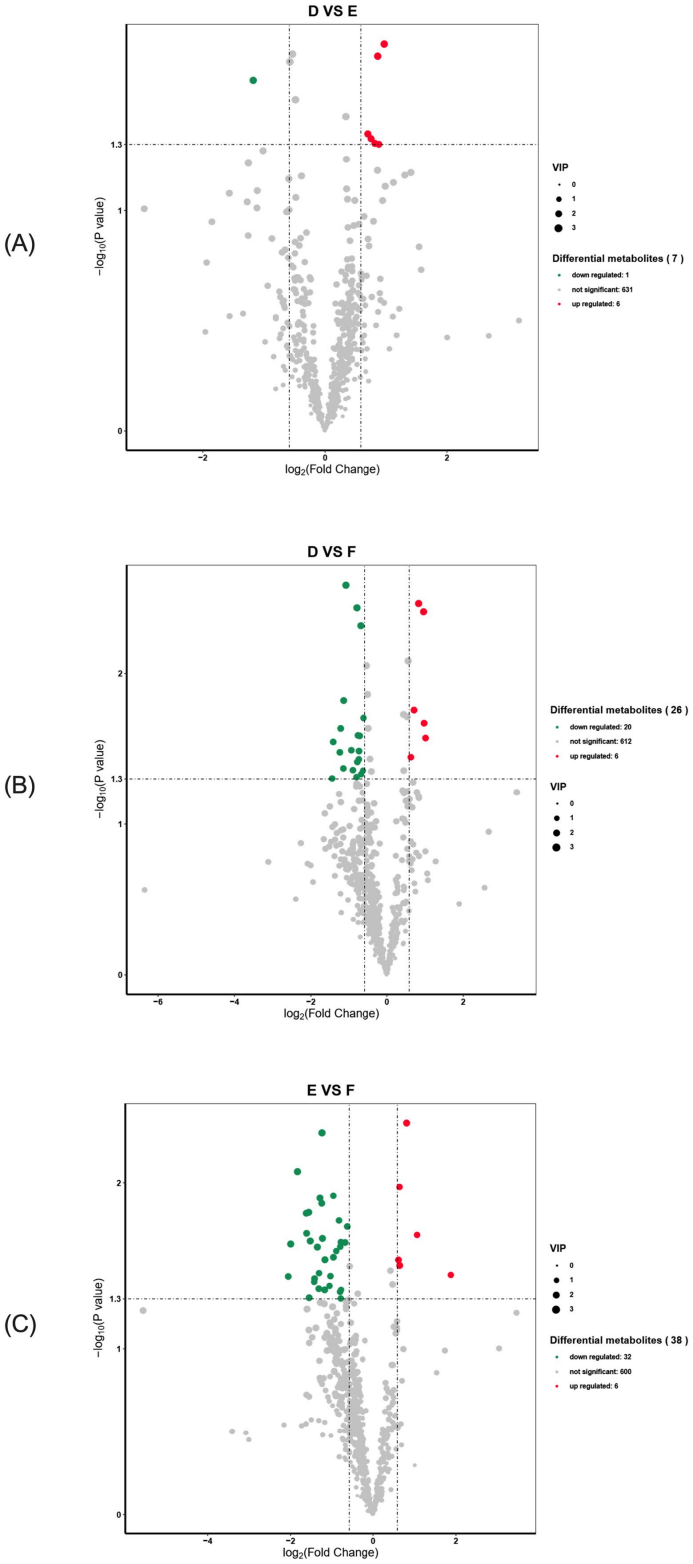
Volcano diagram of differential metabolites in multiparous cattle. **(A)** multiparous cattle control group VS multiparous cattle *L*-Arg group; **(B)** multiparous cattle control group VS Multiparous cattle *L*-Cit group; **(C)** multiparous cattle *L*-Arg group VS Multiparous cattle *L*-Cit.

A total of 638 differential metabolites were detected in serum samples from the multiparous cattle control and *L*-Arg groups, with 7 metabolites showing significant changes. Of these, 1 was down-regulated (*p* < 0.05), 6 were up-regulated (*p* < 0.05), and the remaining 631 metabolites were unaffected (Figure 10A). In the serum samples of the control and *L*-Cit groups, 638 differential metabolites were detected, with 26 showing significant changes. Of these, 20 were down-regulated (*p* < 0.05), 6 were up-regulated (*p* < 0.05), and the remaining 612 metabolites were unchanged (Figure 10B). In serum samples from the *L*-Arg and *L*-Cit groups, 638 differential metabolites were identified, with 38 showing significant changes. Of these, 32 were down-regulated (*p* < 0.05), 6 were up-regulated (*p* < 0.05), and the remaining 600 metabolites exhibited no significant changes (Figure 10C).

**Figure 11:**
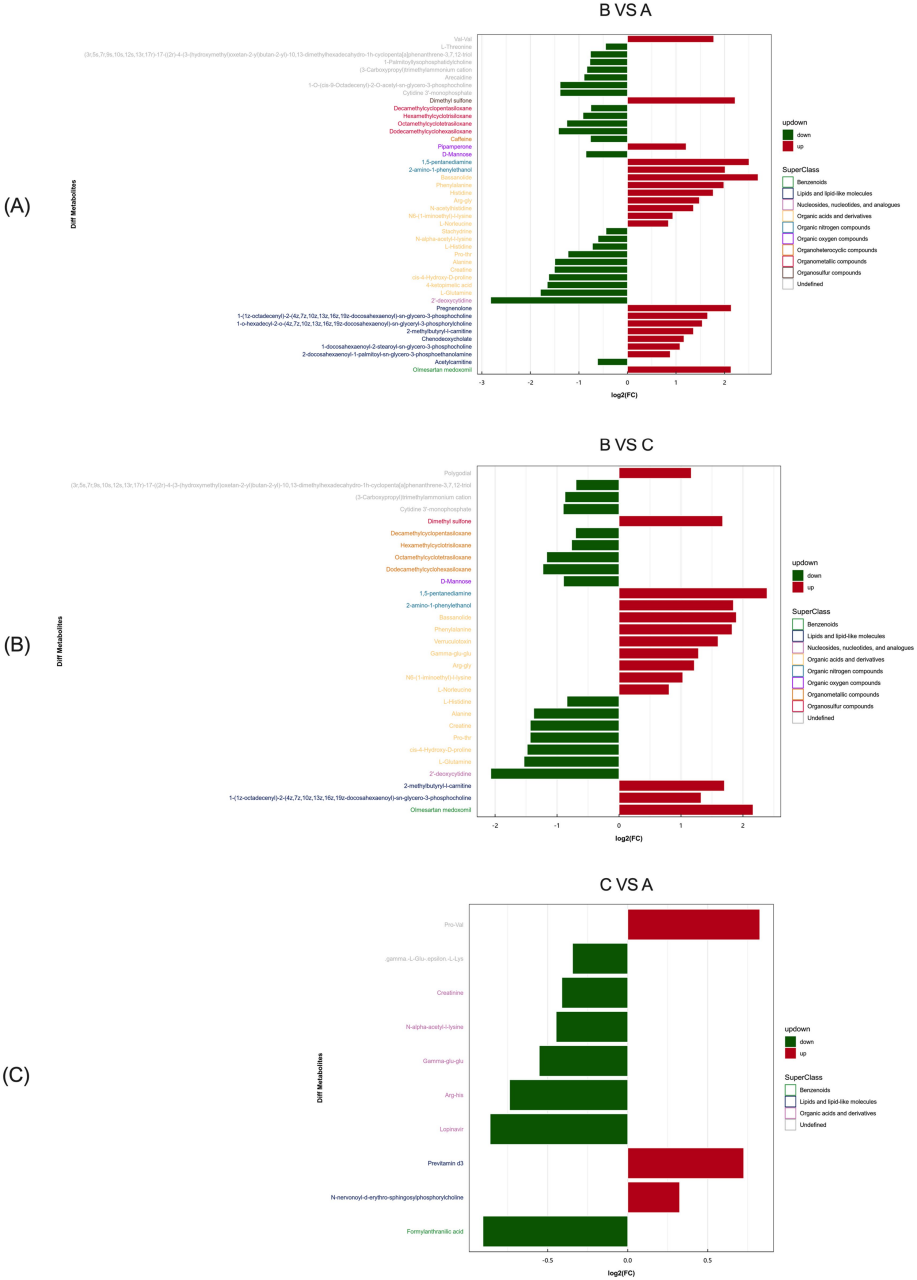
Positive ion mode significance primiparous cattle differential metabolite expression difference fold analysis. **(A)** Primiparous cattle control group VS *L*-Arg group of primiparous cattle; **(B)** Primiparous cattle control group VS *L*-Cit group of primiparous cattle; **(C)** *L*-Arg group of primiparous cattle VS Primiparous cattle *L*-Cit group.

The most significantly enriched differential metabolites in primiparous cattle between the control and *L*-Arg groups included Val-Val, Dimethyl sulfone, Pipamperone, 1,5-pentanediamine, 2-amino-1-phenylethanol, Bassanolide, Phenylalanine, Histidine, Arg-Gly, N-acetylhistidine, N6-(1-iminoethyl)-*L*-lysine, *L*-Norleucine, Pregnenolone, 1-(1z-octadecenyl)-2-(4z,7z,10z,13z,16z,19z- docosahexaenoyl)-sn-glycero-3-phosphoethanolamine, 1-o-hexadecyl-2-o-(4z,7z,10z, 13z,16z,19z-docosahexaenoyl)-sn-glycero-3-phosphoethanolamine, 2-methylbutyryl- *L*-carnitine, Chenodeoxycholate, 1-docosahexaenoyl-2-stearoyl-sn-glycero-3- phosphocholine, 2-docosahexaenoyl-1-palmitoyl-sn-glycero-3-phosphoethanolamine, and Olmesartan medoxomil (Figure 11A).

Between the *L*-Arg and *L*-Cit groups, the most enriched differential metabolites included Polygodial, Dimethyl sulfone, 1,5-pentanediamine, 2-amino-1-phenylethanol, Bassanolide, Phenylalanine, Verruculotoxin, Gamma-glu-glu, Arg-Gly, N6-(1- iminoethyl)-*L*-lysine, *L*-Norleucine, 2-methylbutyryl-*L*-carnitine, 1-(1z-octadecenyl)- 2-(4z,7z,10z,13z,16z,19z-docosahexaenoyl)-sn-glycero-3-phosphoethanolamine, and Olmesartan medoxomil (Figure 11B). In the comparison between the *L*-Cit and control groups, the most enriched differential metabolites were Pro-Val, Previtamin D3, and N-nervonoyl-*D*-erythro-sphingosylphosphorylcholine (Figure 11C).

**Figure 12:**
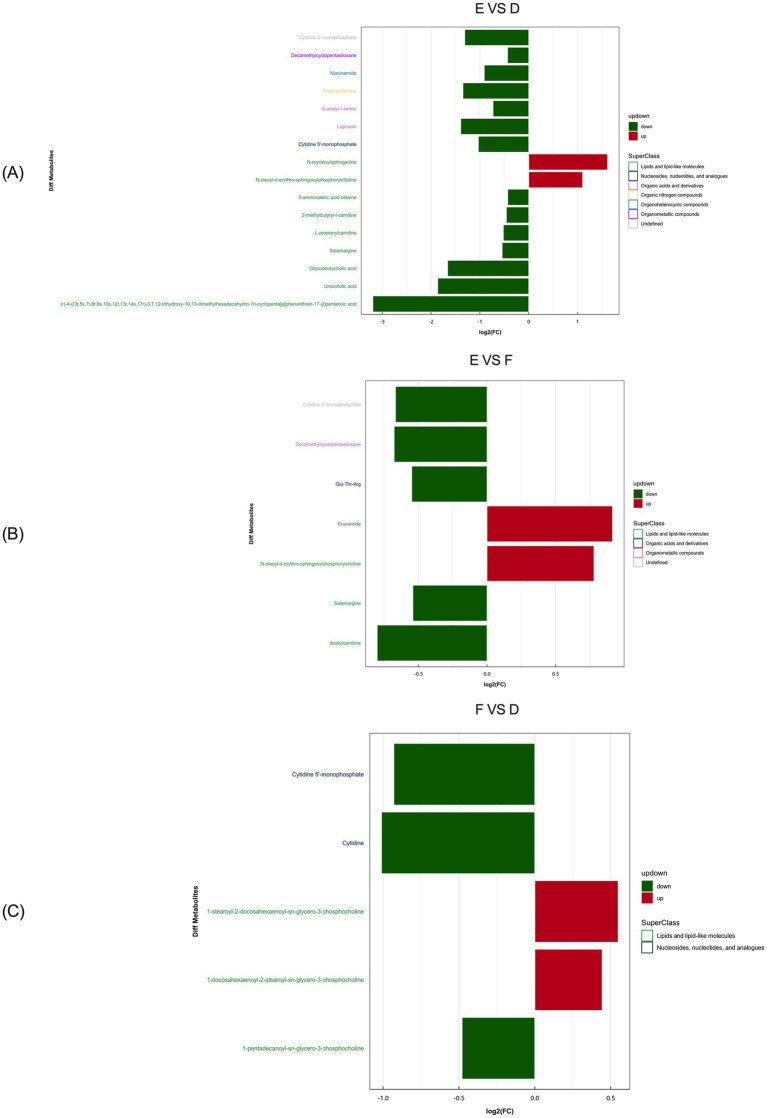
Analysis of multiple differences in the expression of differentially expressed metabolites in multiparous cattle in positive ion mode. **(A)** multiparous cattle control group VS multiparous cattle *L*-Arg group; **(B)** multiparous cattle control group VS Multiparous cattle *L*-Cit group; **(C)** multiparous cattle *L*-Arg group VS Multiparous cattle *L*-Cit.

Based on the multiple changes in differential metabolites between serum samples of the control and *L*-Arg groups, the most significantly enriched metabolites in multiparous cattle were N-myristoylphosphorylcholine and N-oleoyl-*D*-erythro- sphingosylphosphorylcholine (Figure 12A). In comparison between the *L*-Arg and *L*-Cit groups, the most enriched metabolites were Erucamide and N-oleoyl-*D*-erythro- sphingosylphosphorylcholine (Figure 12B). When comparing the *L*-Cit and control groups, the most abundant differential metabolites were 1-stearoyl-2- docosahexaenoyl-sn-glycero-3-phosphocholine and 1-docosahexaenoyl-2-stearoyl-sn- glycero-3-phosphocholine (Figure 12C).

#### 3.6.3 KEGG analysis of metabolite differences between groups

**Figure 13:**
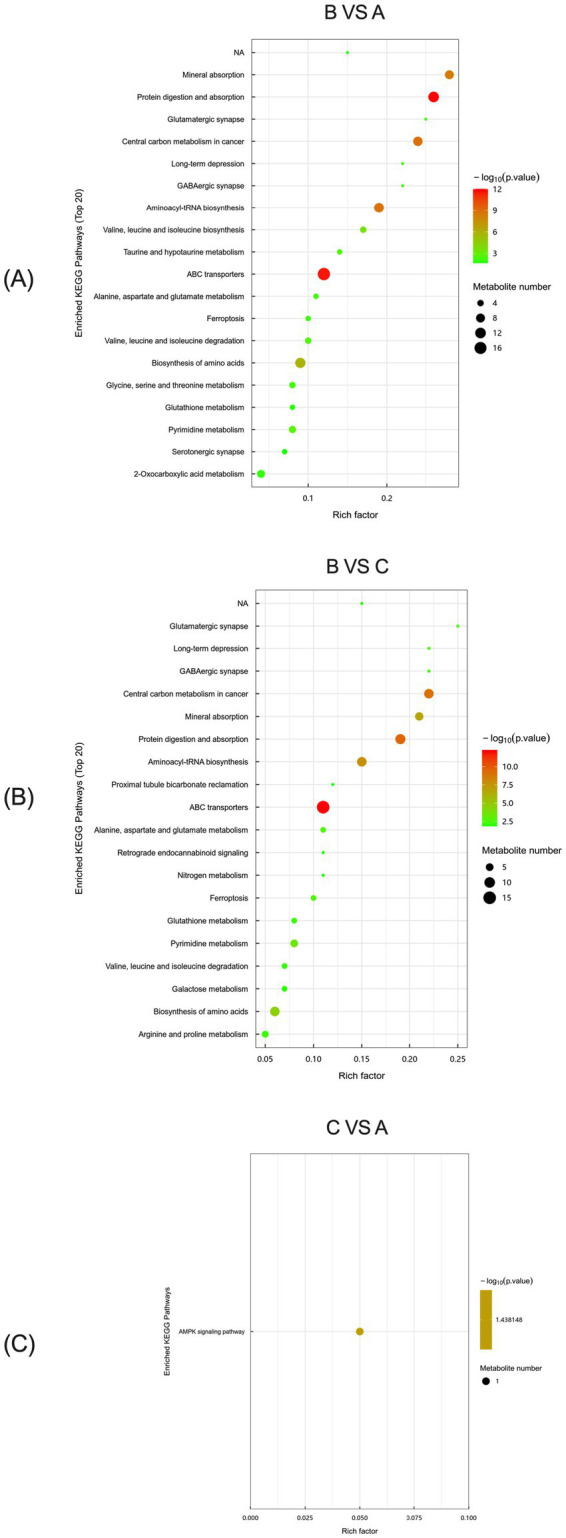
KEGG analysis of serum metabolic differences in primiparous cattle. **(A)** Primiparous cattle control group VS *L*-Arg group of primiparous cattle; **(B)** Primiparous cattle control group VS *L*-Cit group of primiparous cattle; **(C)** *L*-Arg group of primiparous cattle VS Primiparous cattle *L*-Cit group.

KEGG analysis of serum metabolic differences between the control and *L*-Arg groups of primiparous cattle identified two major enrichment pathways: Protein digestion and absorption, and ABC transporters, both being the most significant among the top 20 pathways (Figure 13A). In the comparison between the *L*-Arg and *L*-Cit groups, ABC transporters were the most significant enrichment pathway among the top 20 (Figure 13B). For the control and *L*-Cit groups, KEGG analysis revealed differences in the serum AMPK signaling pathway (Figure 13C).

**Figure 14:**
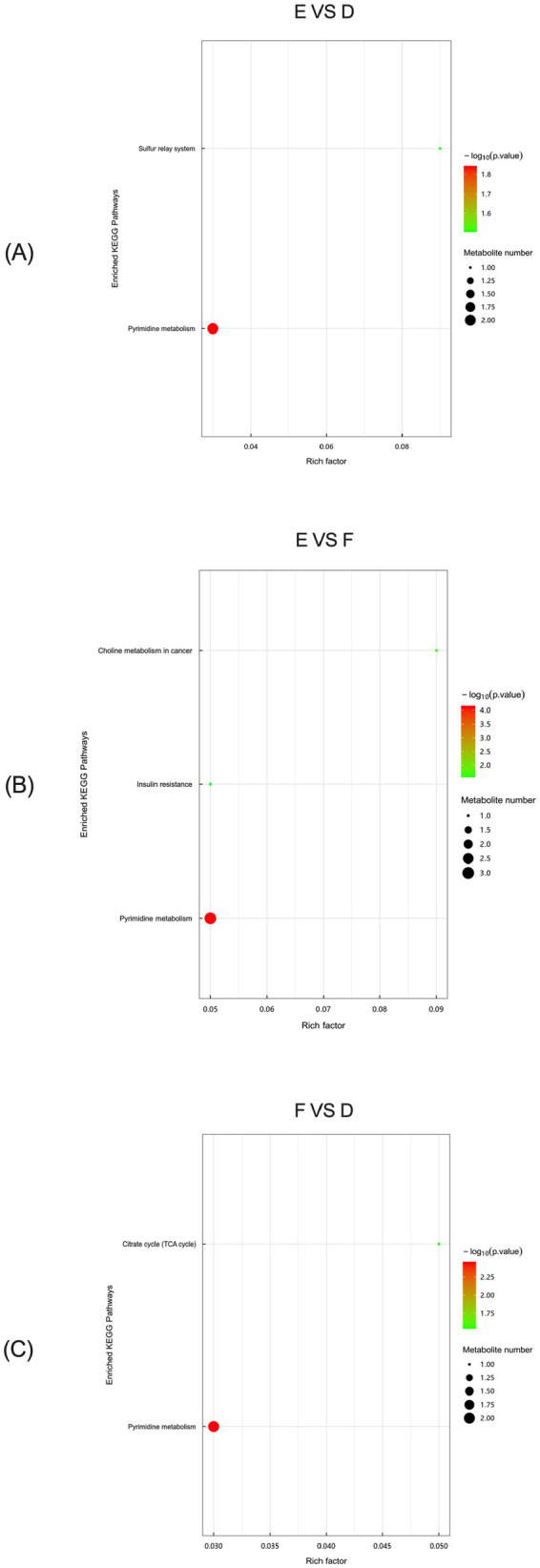
KEGG analysis of metabolic differences in multiparous bovine serum. **(A)** multiparous cattle control group VS multiparous cattle *L*-Arg group; **(B)** multiparous cattle control group VS Multiparous cattle *L*-Cit group; **(C)** multiparous cattle *L*-Arg group VS Multiparous cattle *L*-Cit.

In multiparous cattle, KEGG analysis of serum metabolic differences between the control and *L*-Arg groups showed the Pyrimidine metabolism pathway to be the most significant among the two major enrichment pathways (Figure 14A). Similarly, between the *L*-Arg and *L*-Cit groups, the Pyrimidine metabolism pathway was the most significant of the three main enrichment pathways (Figure 14B). Furthermore, KEGG analysis of serum metabolic differences between the control and *L*-Cit groups in multiparous cattle confirmed the Pyrimidine metabolism pathway as the most significant of the two major enrichment pathways (Figure 14C).

#### 3.6.4 Correlation analysis between serum metabolites and blood indexes

**Figure 15:**
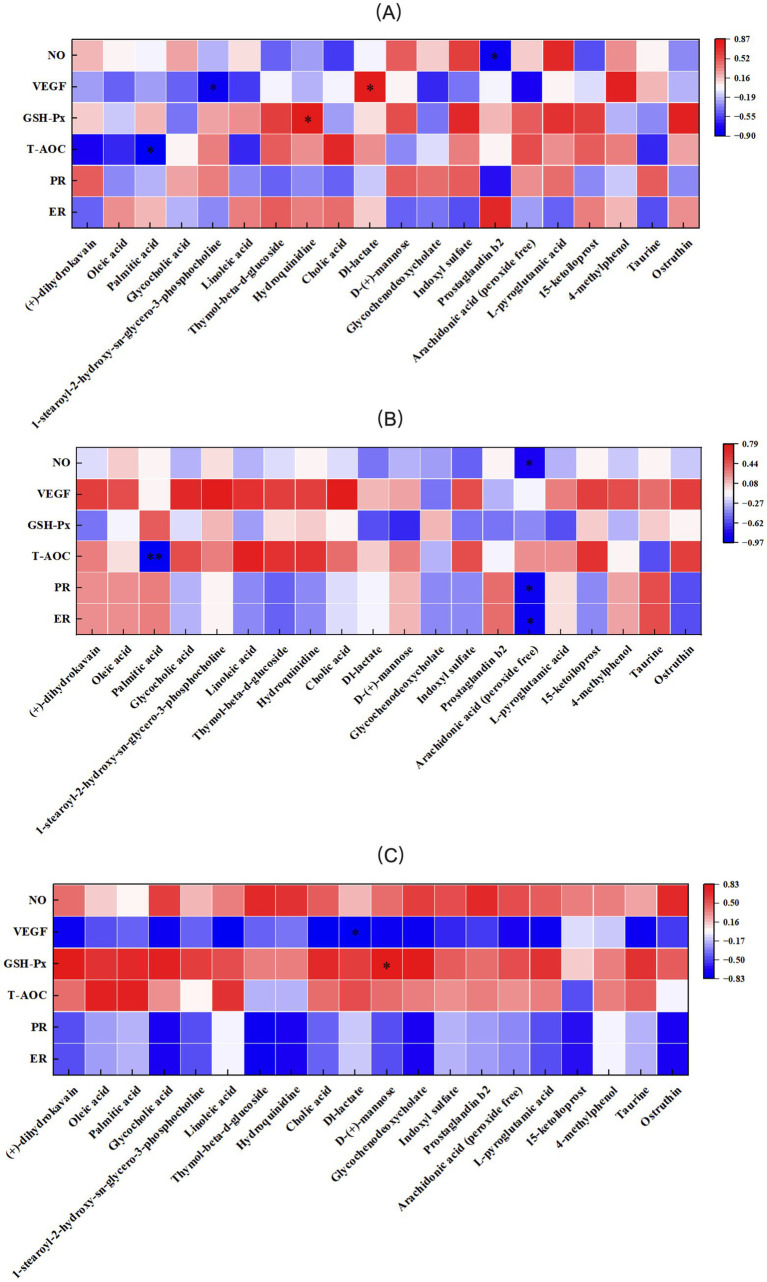
Correlation analysis of traits and metabolites in primiparous cattle. **(A)** Primiparous cattle control group VS *L*-Arg group of primiparous cattle; **(B)** Primiparous cattle control group VS *L*-Cit group of primiparous cattle; **(C)** *L*-Arg group of primiparous cattle VS Primiparous cattle *L*-Cit group.

**Figure 16:**
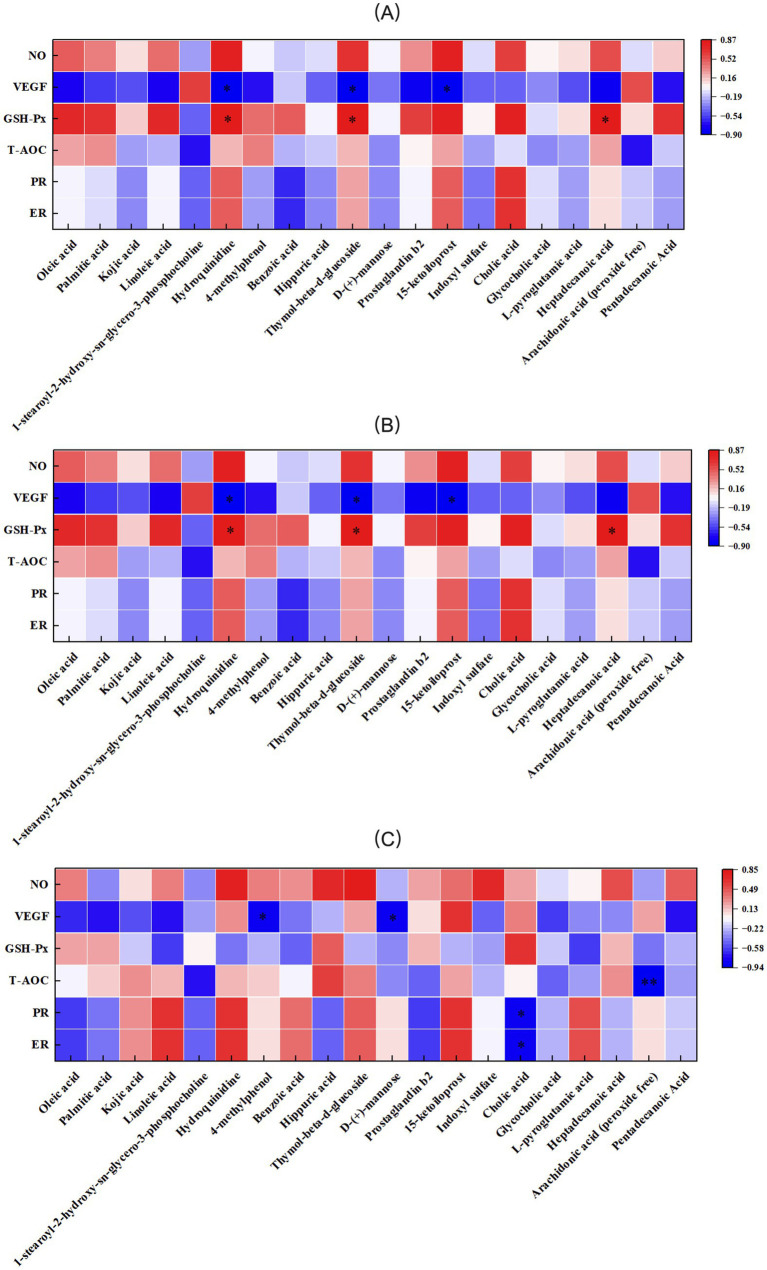
Correlation analysis between traits and metabolites in multiparous cattle. **(A)** multiparous cattle control group VS multiparous cattle *L*-Arg group; **(B)** multiparous cattle control group VS Multiparous cattle *L*-Cit group; **(C)** multiparous cattle *L*-Arg group VS Multiparous cattle *L*-Cit.

The correlation between antioxidant indices, VEGF, PR, ER, and serum metabolic differences in primiparous cattle was analyzed. A significant negative correlation was observed between NO and Prostaglandin B2 in the control group. VEGF exhibited a negative correlation with 1-stearoyl-2-hydroxy-sn-glycero-3- phosphocholine and a positive correlation with Dl-lactate. GSH-Px was positively correlated with Hydroquinidine, while T-AOC showed a significant negative correlation with Palmitic acid (Figure 15A). In the *L*-Arg group, T-AOC was significantly negatively correlated with Palmitic acid, and NO, PR, and ER were significantly negatively correlated with Arachidonic acid (peroxide free; Figure 15B). In the *L*-Cit group, VEGF was significantly negatively correlated with Dl-lactate, and GSH-Px was significantly positively correlated with D-(+)-mannose (Figure 15C). The correlation analysis of antioxidant indices, VEGF, PR, ER, and serum metabolic differences in multiparous cattle revealed that T-AOC was significantly negatively correlated with 15-ketoiloprost in the control group. NO exhibited a negative correlation with Cholic acid and a positive correlation with *L*-pyroglutamic acid (Figure 16A). In the *L*-Arg group, GSH-Px showed significant positive correlations with Hydroquinidine, Thymol-beta-d-glucoside, and Heptadecanoic acid, while VEGF was significantly negatively correlated with Hydroquinidine, Thymol-beta-d-glucoside, and 15-ketoiloprost (Figure 16B). In the *L*-Cit group, VEGF was negatively correlated with 4-methylphenol and D-(+)-mannose, while T-AOC showed a significant negative correlation with Arachidonic acid (peroxide free). Furthermore, ER and PR were significantly negatively correlated with Cholic acid (Figure 16C).

## 4 Discussion

Nutritional levels are closely linked to the estrous cycles of female animals, influencing the follicular microenvironment, reducing follicular energy levels, and inhibiting follicle growth and oocyte maturation, which can lead to anovulation and extended re-pregnancy intervals (10). The addition of exogenous nutrients can enhance the uterine environment and improve endometrial health, thereby increasing conception rates. As a non-essential amino acid, *L*-Cit is converted into *L*-Arg and NO by the enzymes argininosuccinate synthase and argininosuccinate lyase (11). The NO produced from *L*-Cit can increase vascular permeability, promote placental development, and provide nutrients essential for embryo implantation (12). Before ovulation, NO helps regulate ovarian blood circulation by inhibiting vasoconstrictors. Proper vascularization is crucial for the selection, growth, and maturation of follicles in female animals, and increased blood flow to dominant follicles directly influences conception rates (13). Supplementing *L*-Arg in females with irregular estrous cycles and poor ovarian function enhances ovarian blood supply (14). In ovine estrus synchronization, *L*-Arg supplementation alleviates delayed estrus due to nutritional restrictions, stimulates follicular development, increases ovulation, and significantly improves conception rates (15, 16). Vander found that adding 1.0% *L*-Arg between days 14 and 30 of gestation increased pregnancy rates and litter size by 15 and 8%, respectively (17). In this experiment, the estrus and conception rates of cows injected intraperitoneally with *L*-Arg and *L*-Cit were significantly higher than those of the control group, aligning with previous findings. In cases of poor maternal nutrition, short-term supplementation with *L*-Arg and *L*-Cit significantly boosts the conception rates of estrous cows. Maternal nutrition during pregnancy, along with the uterine microenvironment, plays a pivotal role in follicular development and embryo survival (18). Ensuring adequate nutrient supply, particularly amino acids, during the mating period is critical for successful follicular development and embryo viability (19).

Animal reproduction is regulated by numerous hormones that directly influence estrus, ovulation, embryo implantation, pregnancy, and parturition. In this study, intraperitoneal injection of *L*-Arg in primiparous cows significantly increased serum LH levels on the 1st and 8th days. In multiparous cattle, the E2 level was significantly reduced on the 1st, 8th, and 10th days after intraperitoneal injection of *L*-Arg and *L*-Cit, compared to the control group. Bonavera found that *L*-Arg injection increased both the concentration and duration of LH induced by E2 in mice (20). Zeitoun supplemented 75 and 150 mg/kg *L*-Arg in the diets of ewes during early and late pregnancy, respectively, and observed that both *L*-Arg concentrations reduced plasma E2 levels in early pregnancy (21). The results for LH and E2 in this experiment were consistent with previous findings. Furthermore, the levels of FSH and GnRH on the 1st and 10th days post-injection of *L*-Cit were significantly lower than those in the control group. The P level was significantly higher than that of the control group on the 8th and 21st days following *L*-Arg and *L*-Cit injection. Saevre’s study reported that daily injections of 27 mg/kg body weight arginine hydrochloride to ewes in early pregnancy significantly reduced plasma P levels (22). The results for FSH, GnRH, and P in this experiment were inconsistent with prior studies, likely due to differences in animal breeds or the timing of *L*-Arg and *L*-Cit supplementation. Both *L*-Cit and *L*-Arg can significantly increase NO levels in the body through the Cit-Arg or Arg-NO cycle (23). NO plays a pivotal role in neuroendocrine regulation by promoting the release of GnRH from the hypothalamus, which directly stimulates LH and FSH secretion from the anterior pituitary (4). Additionally, NO regulates the expression of steroid synthase in follicles, thereby influencing the normal secretion of E2 and P. Low levels of P and E2 stimulate estrus in female animals, while high levels of P inhibit estrus (24).

The imbalance between the maternal placenta’s ability to transport oxygen and nutrients and the fetus’s needs can lead to a reduction in embryo numbers and a delay in fetal growth. Supplementing *L*-Arg during pregnancy can mitigate embryo loss and enhance fetal development, as this amino acid plays a key role in metabolic pathways for the formation of active molecules such as polyamines and NO (25). These processes are crucial for embryonic development, embryogenesis, uterine function during pregnancy, growth, development, and fetal survival (26, 27). Dietary supplementation with *L*-Arg in various animal species has been shown to promote embryo survival rates. For instance, adding 1% arginine-HCl to the pre-pregnancy diet improves fetal survival in sows (28). Intravenous *L*-Arg injections in high-yielding ewes during pregnancy reduce fetal mortality and promote fetal growth (5). Additionally, *L*-Arg supplementation in the diet of pregnant sows significantly reduces neonatal piglet mortality (29). In this experiment, the embryo apoptosis rate in the *L*-Arg and *L*-Cit groups of primiparous cows decreased by 36 and 22.5%, respectively, from days 21 to 42 compared to the control group. In multiparous cows, the embryo apoptosis rate in the *L*-Arg and *L*-Cit groups decreased by 11.9 and 22.7%, respectively, during the same period. These results indicate that intraperitoneal injection of *L*-Arg and *L*-Cit can promote early embryo development and implantation, reducing early embryo apoptosis. NO plays a pivotal role by dilating the capillaries in the uterus and placenta, enhancing blood and oxygen supply to the placenta, and accelerating nutrient delivery to the fetus, thereby supporting normal embryo development and implantation (30). Crane’s research demonstrated that *L*-Arg supplementation improved early embryo survival in ewes (31), and Zeng’s study in rats found that a 1.3% *L*-Arg diet during pregnancy increased embryo implantation points by 29% on day 7, as well as increasing litter size and live birth rate by 30% (32). In the present study, intraperitoneal injection of *L*-Arg and *L*-Cit reduced embryonic apoptosis, likely due to enhanced synthesis of NO and polyamines. *L*-Cit, an endogenous precursor of *L*-Arg, is converted to *L*-Arg by argininosuccinate synthase and lyase. Furthermore, *L*-Cit provides substrates for the synthesis of polyamine molecules and NOS to produce NO (33). This process effectively reduces early embryo mortality and improves embryo survival rates.

Oxidative stress refers to the imbalance between free radicals and the antioxidant defense systems in the body (34). During pregnancy, if oxidative stress is not properly regulated, it can lead to changes in cell composition, jeopardizing the health of both female animals and their fetuses (35). T-AOC reflects the overall antioxidant capacity in animals, with its levels indicating the animal’s health status. SOD, CAT, and GSH-Px possess the ability to scavenge free radicals, while MDA levels indicate the extent of free radical-induced cellular damage (36). SOD decomposes toxic O2- into less harmful H2O2, which can further generate hydroxyl (OH) radicals. CAT and GSH-Px convert H2O2 into water, helping to neutralize free radicals (37). Supplementing arginine in the diet of rats significantly increases serum levels of T-AOC, GSH-Px, and SOD (38). Morris’s study demonstrated that adding 20 g of rumen-protected arginine to the diet of pregnant ewes significantly increased the activity of T-AOC and SOD in their plasma (39). In this experiment, after intraperitoneal injection of *L*-Arg and *L*-Cit, there was a significant increase in serum concentrations of T-AOC, SOD, and GSH-Px in cows. The concentration of CAT decreased significantly, while MDA levels did not change significantly. Correlation analysis between the differential phenotype of primiparous cows and their serum metabolic differences revealed that in the *L*-Cit group, GSH-Px was significantly positively correlated with D-(+)-mannose. In multiparous cows, GSH-Px was significantly positively correlated with Hydroquinidine, Thymol-beta-d-glucoside, and Heptadecanoic acid in the *L*-Arg group. These findings indicate that intraperitoneal injection of *L*-Arg and *L*-Cit enhanced the antioxidant capacity of cows. After *L*-Arg injection, a significant negative correlation between T-AOC and Palmitic acid was observed in the serum of primiparous cows. Similarly, following *L*-Cit injection, a significant negative correlation between T-AOC and Arachidonic acid (peroxide free) was found in the serum of multiparous cows. These changes may result from the stress induced by intraperitoneal injection, which could lead to decreased antioxidant enzyme activity in the cows.

Plasma is a commonly used sample type in metabolomics research, as it reflects the overall metabolic state of the body and provides valuable insights into nutrient absorption, energy utilization, and health status. ABC transporters, a superfamily of membrane proteins with diverse functions, are present in both prokaryotes and eukaryotes. They utilize the energy generated by ATP hydrolysis to transport various substances across membranes (40–42). ABC transporters are integral to many physiological functions in the body, with nearly all molecules involved in these processes serving as substrates for ABC transport (43). These transporters play a critical role in early embryonic development, particularly during the cleavage and blastocyst stages. They regulate material metabolism and signal transduction in embryos, thereby creating the necessary conditions for normal cell division and differentiation (44). In the present study, the rate of early embryo apoptosis in cows decreased following intraperitoneal injection of *L*-Arg and *L*-Cit. This may be attributed to the impact of *L*-Arg and *L*-Cit on the synthesis and activity of ABC transporters, which in turn affects their expression and function, ultimately reducing early embryo apoptosis.

Proteins, essential biological macromolecules, perform a wide range of functions in the body. Amino acids, the basic building blocks of proteins, are vital for various physiological processes. In this study, the plasma concentrations of most amino acids in primiparous cows increased following intraperitoneal injection of *L*-Arg and *L*-Cit. This corresponds with the protein digestion and absorption pathways, indicating that *L*-Arg and *L*-Cit enhance protein digestion and absorption in cows, thereby potentially improving reproductive performance. Phenylalanine, an essential aromatic amino acid, must be obtained through diet and is primarily metabolized in the liver, where it is converted into tyrosine by phenylalanine hydroxylase. Tyrosine plays an important role in cognitive and executive functions (45). Dopamine, an intermediate product of phenylalanine metabolism, regulates the secretion of gonadotropin-releasing hormone (GnRH) through dopaminergic neurons. In turn, GnRH influences the release of follicle-stimulating hormone (FSH) and luteinizing hormone (LH), which regulate follicular development, ovulation, and estrus. During pregnancy, dopamine plays a pivotal role in maintaining pregnancy by regulating endometrial receptivity and promoting embryo implantation. Additionally, dopamine influences placental development and function, including the regulation of placental angiogenesis and nutrient transport (46). The changes in ornithine, valine, and other amino acids observed in the serum of cows in this experiment may be closely associated with the conversion of *L*-Cit to arginine and the subsequent metabolic processes of arginine. Ornithine, an important intermediate in arginine metabolism, may affect polyamine synthesis (47), which in turn influences embryonic development. In this study, intraperitoneal injection of *L*-Arg and *L*-Cit increased the estrus and conception rates of cows and reduced the early embryo apoptosis rate, likely due to the conversion of *L*-Cit to arginine and its metabolic processes.

Reproduction is a high-energy-demanding process that occurs only when mammals have sufficient available energy. AMPK, the key regulator of cellular energy homeostasis, is closely linked to follicular development, granulosa cell proliferation, and pregnancy regulation (48). Studies have shown that conditional loss of AMPKα in the mouse uterus results in failure of scarless endometrial regeneration after delivery and severe endometrial fibrosis, leading to embryo implantation failure and reduced fertility (49). Furthermore, AMPK activation promotes uterine artery vasodilation, suggesting that it helps maintain uterine placental blood flow and ensures normal pregnancy (50). AMPK is also essential for embryonic growth and differentiation, with double knockout of PRKAA1 and PRKAA2 in mice causing embryonic death around day 10.5 of pregnancy (51). *L*-Arg and *L*-Cit participate in the urea cycle and other nitrogen metabolism processes, influencing amino acid metabolism balance and cellular energy status. They also regulate cell energy metabolism by affecting nitric oxide (NO) synthesis (52). When cellular energy demands increase or energy levels decline, *L*-Arg stimulates NO production, which activates AMPK. This initiates a cascade of reactions to maintain cellular energy homeostasis, promoting catabolic pathways to generate more energy and inhibiting anabolic pathways to conserve energy. This process ensures that cows receive adequate energy support during reproduction, enhancing reproductive performance. Pyrimidine nucleotides are essential for the synthesis of DNA, RNA, glycoproteins, and phospholipids, and are involved in cell metabolism and proliferation (53). In this experiment, intraperitoneal injection of *L*-Arg significantly enriched the AMPK and Pyrimidine metabolism pathways in the serum of primiparous cows. This suggests that *L*-Arg injection affected the balance of amino acid metabolism and cellular energy status, thereby improving the cows’ conception rate. Metabolomic analysis suggested potential associations of *L*-Arg with the protein digestion and absorption and ABC transporters pathways, and *L*-Cit with the ABC transporters pathway, in primiparous cows; both amino acids showed putative synergistic effects on the pyrimidine metabolism pathway in multiparous cows. These pathways are plausibly involved in reproduction and cellular energy balance, but their functional roles require further validation.

**Figure 17:**
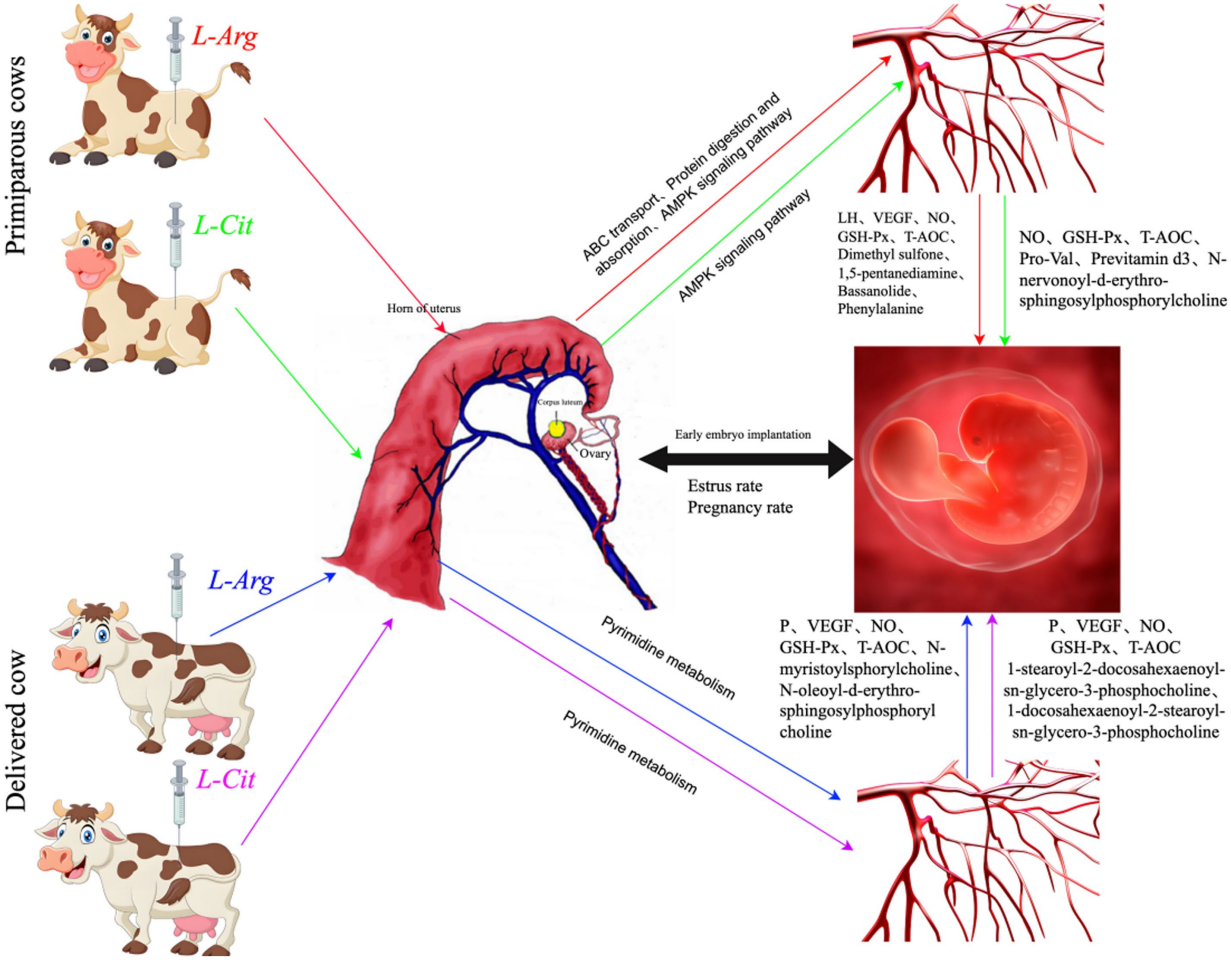
The possible mechanism change the picture of intraperitoneal injection of *L*-Arg and *L*-Cit affecting the reproductive performance of cows.

*L*-Cit can be converted into *L*-Arg through a series of enzymatic reactions in the kidneys and other tissues of female animals. In intestinal cells, glutamine and proline are converted into citrulline, which is then transported to the kidneys where it is synthesized into arginine *via* the ornithine cycle (54). Arginine is further metabolized to produce NO, polyamines, urea, and other metabolites. NO plays a key role in regulating uterine placental blood flow and preventing intrauterine growth restriction, both of which are vital for the normal development and maturation of embryos. Polyamines are essential for cell proliferation and differentiation (30). As a precursor for the synthesis of NO and polyamines, arginine supports the rapid growth of the placenta during early pregnancy in mammals (55). In this experiment, intraperitoneal injection of *L*-Arg significantly increased the serum levels of LH, VEGF, NO, GSH-Px, T-AOC, Dimethyl sulfone, 1,5-pentane,diamine, Bassanolide, and Phenylalanine in primiparous cows compared to the control group. In multiparous cows, serum levels of P, VEGF, NO, GSH-Px, T-AOC, N-myristoylsphorylcholine, and N-oleoyl-*D*-erythro-sphingosylphosphorylcholine were also significantly higher than in the control group. After intraperitoneal injection of *L*-Cit, NO, GSH-Px, T-AOC, Pro-Val, Previtamin D3, and N-nervonoyl-*D*-erythro- sphingosylphosphorylcholine levels in the serum of primiparous cows were significantly elevated compared to the control group. In multiparous cows, serum levels of P, VEGF, NO, GSH-Px, T-AOC, 1-docosahexaenoyl-2-docosahexaenoyl-sn- glycero-3-phosphocholine, and 1-docosahexaenoyl-2-docosahexaenoyl-sn-glycero-3- phosphocholine were also significantly higher than those of the control group. Furthermore, after intraperitoneal injection of *L*-Arg and *L*-Cit, the conception rate of both primiparous and multiparous cows increased. This is likely due to the fact that *L*-Arg and *L*-Cit injections enhanced antioxidant enzyme activity in the body, improved protein digestion and absorption, and increased uterine placental blood flow regulation. These effects likely promoted early embryo implantation, leading to an increase in the conception rate of the cows (Figure 17). L-Arg and *L*-Cit were associated with higher pregnancy rates at days 21–42 and lower inferred embryo loss rates in both primiparous and multiparous cows. This association may be attributed to increased levels of NO, GSH-Px, T-AOC, and VEGF (which support vascularization and antioxidant defense critical for embryo survival), though direct evidence of enhanced implantation was not measured.

## 5 Conclusion

This study is the first to demonstrate that *L*-Arg and *L*-Cit regulate cow reproduction via distinct pathways in primiparous vs. multiparous cows, and their synergistic effect on pyrimidine metabolism in multiparous cows. Under the conditions of this experiment, *L*-Arg improves primiparous reproduction via protein digestion/ABC transporters; *L*-Cit acts via ABC transporters. Both synergistically activate pyrimidine metabolism in multiparous cows. *L*-Cit is more effective (multiparous conception rate: 93.3% vs. 80.0% for *L*-Arg, *p* < 0.05). This study was conducted in Simmental cows in Xinjiang; the results may not apply to other breeds. Further studies should detect endometrial receptivity and placental histology to clarify the mechanisms of colonization.

## Data Availability

The original contributions presented in the study are included in the article/supplementary material, further inquiries can be directed to the corresponding author.
